# Overexpression of BDNF Increases Excitability of the Lumbar Spinal Network and Leads to Robust Early Locomotor Recovery in Completely Spinalized Rats

**DOI:** 10.1371/journal.pone.0088833

**Published:** 2014-02-14

**Authors:** Ewelina Ziemlińska, Sebastian Kügler, Melitta Schachner, Iwona Wewiór, Julita Czarkowska-Bauch, Małgorzata Skup

**Affiliations:** 1 Nencki Institute of Experimental Biology, Warsaw, Poland; 2 Center of Molecular Physiology of the Brain, University of Göttingen, Göttingen, Germany; 3 Center for Neuroscience, Shantou University Medical College, Shantou, China; Emory University, United States of America

## Abstract

Strategies to induce recovery from lesions of the spinal cord have not fully resulted in clinical applications. This is a consequence of a number of impediments that axons encounter when trying to regrow beyond the lesion site, and that intraspinal rearrangements are subjected to. In the present study we evaluated (1) the possibility to improve locomotor recovery after complete transection of the spinal cord by means of an adeno-associated (AAV) viral vector expressing the neurotrophin brain-derived neurotrophic factor (BDNF) in lumbar spinal neurons caudal to the lesion site and (2) how the spinal cord transection and BDNF treatment affected neurotransmission in the segments caudal to the lesion site. BDNF overexpression resulted in clear increases in expression levels of molecules involved in glutamatergic (VGluT2) and GABAergic (GABA, GAD65, GAD67) neurotransmission in parallel with a reduction of the potassium-chloride co-transporter (KCC2) which contributes to an inhibitory neurotransmission. BDNF treated animals showed significant improvements in assisted locomotor performance, and performed locomotor movements with body weight support and plantar foot placement on a moving treadmill. These positive effects of BDNF local overexpression were detectable as early as two weeks after spinal cord transection and viral vector application and lasted for at least 7 weeks. Gradually increasing frequencies of clonic movements at the end of the experiment attenuated the quality of treadmill walking. These data indicate that BDNF has the potential to enhance the functionality of isolated lumbar circuits, but also that BDNF levels have to be tightly controlled to prevent hyperexcitability.

## Introduction

Mechanisms underlying the improvement of motor abilities after spinal cord injury are still a matter of debate; brain-derived neurotrophic factor (BDNF) is considered an important player [1]–[3]. To generate stepping, lumbar spinal circuitries have to adapt to the loss of supraspinal inputs [4]. After a complete spinal cord transection, this adaptation involves functional reorganization as demonstrated at the behavioral, biochemical, structural, and electrophysiological levels [5]–[10]. However, it is still not known to which extent changes in neurotransmitter levels contribute to this reorganization. Within minutes extracellular glutamate (Glu) and aspartate (Asp) levels are increased and contribute to neuronal damage [11], [12]. Within hours tissue Glu and Asp as well as GABA and glycine (Gly) levels in injured spinal cord decrease, but GABA and Gly recover thereafter [13], [14]. An increase in Glu levels and GABA high-affinity uptake in spastic paraplegic dogs one month after spinal cord injury [15], was attributed to axonal sprouting of primary sensory neurons [16], [17] and interneurons [18]. Neonatal rats with spinal cord transection also showed adaptations of excitatory and inhibitory circuits [19]. In adult chronic spinal rats less GABAergic inhibition than in acute spinal rats was reported [20]. In adult spinal cats, an increase of GABA-mediated inhibition in the lumbar spinal circuits [21] and of GABA synthesizing enzyme glutamate decarboxylase 67 (GAD67) levels [6] were observed. A prevailing view thus emerged that in adult animals spinal cord injury leads to a loss of balance between excitatory and inhibitory systems that leads to inappropriate locomotion [22].

Neurotrophins have been shown to influence the establishment of neural networks in development and regeneration. Among these, BDNF is important for neurite outgrowth, synaptogenesis as well as synaptic transmission and synaptic plasticity [23], [24]. Both, BDNF delivery and/or sensorimotor training, which elevates spinal cord levels of BDNF [2], [25]–[30], improve stepping behavior after spinal transection by augmenting the plasticity of lumbar spinal networks and efficacy of sensorimotor pathways. In adult rats with spinal cord injury, these treatments normalize the levels of cyclic AMP responsive element binding protein (CREB) and synapsin 1 [2], [31], and result in axonal regrowth/sprouting and possibly sparing of severed axons and the extent of synaptic [32], [33] inputs to lumbar motoneurons [2], [28], [34]. Also cellular electrophysiological parameters are changed after BDNF delivery or training, increasing motoneuron susceptibility to discharge [8], [35], [36] and activating lumbar interneurons implicated in improved stepping functions [36]. These changes are most likely related to the contribution of BDNF to regulation of the neuron-specific potassium-chloride co-transporter (KCC2) [24], [36]–[38] maintaining low Cl^-^ intracellular concentrations which determine the extent of inhibition by GABA and glycine [24], [39]–[42]. With acute administration of BDNF up-regulating spinal levels of plasmalemmal KCC2 in spinal rats [43], available data suggest that BDNF alters the levels of presynaptic and postsynaptic systems caudal to the transection site.

To clarify how the complete spinal transection at the low thoracic segments in adult rats affects: (1) the levels of excitatory and inhibitory amino acids in the spinal cord and (2) the segmental distribution and levels of these amino acids and related molecules in the spinal cord with elevated and sustained levels of BDNF (delivered via AAV with the neuronal promoter to the L1–2 segments at the time of placing the lesion), these markers were measured rostrally and caudally to the transection site. We took into account the functional and biochemical differences among lumbar segments in the adult rat, where greater capacity to induce the locomotor pattern is attributed to the rostral (L1–2) than to the caudal lumbar segments (L3–6), where the motoneurons innervating the majority of hindlimb muscles are located (for review see [44]–[47]).

We here show that maintaining elevated levels of BDNF in the lumbar spinal cord deprived of descending inputs over the seven weeks results in improvement of locomotor function during the second week after injury, in parallel with increased glutamatergic and GABAergic neurotransmission, that had been decreased by the lesion. Strongly elevated levels of GABA, GAD67, GAD65 and moderately elevated levels of glutamate vesicular transporter 2 (VGluT2) expression above control found in the caudal segments indicate an altered balance of excitatory and inhibitory neurotransmission, developing in time after injury. Increased GABA levels in conjunction with sustained reduced KCC2 expression in AAV-BDNF treated animals may underlie hyperactivity of motoneurons, resulting in episodes of clonic movements, which appeared at later time points after spinalization, indicating the importance of time-controlled application of BDNF after central nervous system injury.

Preliminary accounts of this work have been presented elsewhere [48]–[50].

## Materials and Methods

### Chemicals

All chemicals were purchased from Sigma-Aldrich (St. Louis, MO, USA), except for ARL 67156, CGS 21680, and PSB-36 (Tocris Bioscience, Ellisville, MO, USA), 1-octanesulfonic acid sodium salt and Mowiol (81381) (Fluka, Steinheim, Germany), and Na_2_HPO_4_, NaH_2_PO_4_ and CH_3_COONa (POCh, Gliwice, Poland).

### Primary Antibodies

cMYC #2276, 1∶1000, Phospho-TrkA (Y490)/TrkB (Y516)(G35G9), 1∶1000, Phospho-MAPK (T202/T204)(E10), 1∶2500, and Phospho-PLCγ1 (Y783), 1∶1000 (all from Cell Signaling Tech., Danvers, MA, USA); Phospho-TrkB (Y705), 1∶500 (Abcam, Cambridge, UK); VAChT, 1∶5000 (Sigma-Aldrich); BDNF (N-20), 1∶1000 and TrkB (sc-12), 1∶1000 (Santa-Cruz Technologies, Santa Cruz, CA, USA); β-tubulin clone TUB2.1, 1∶10 000 (Sigma-Aldrich); β-actin, 1∶1000 (IMGENEX, San Diego, CA, USA); GAD67 #MAB5406, 1∶1000 and KCC2 #07–432, 1∶1000 (Millipore, Billerica, MA, USA).

### Cloning of the BDNF Coding Sequence into the AAV Plasmid (pAAV) Vector’s Genome and Functional Validation of AAV-expressed BDNF in Primary Cultures

Full length rat pre-pro-BDNF was cloned into AAV vector genomes with neuron-specific human synapsin 1 gene promoter (SYN). Vectors expressing EGFP were used as controls (AAV1/2-SYN-EGFP) [51]. Recombinant AAV vectors of the hybrid serotype 1/2 (AAV1/2-SYN-BDNF) were produced essentially as described before [52]. To verify that viral vector-mediated expression of cMYC-tagged BDNF resulted in appropriate TrkB signaling, we transduced neurons in primary cortico-hippocampal cell cultures (DIV6), obtained as described previously [53], with 0.5×10^8^–1.0×10^8^ - transduction units of AAV1/2-SYN-BDNF per well to express BDNF (**Figure S1A**). The onset of the resulting BDNF secretion was fast, increasing rapidly between the 2^nd^ and 4^th^ days post-transduction (DIV8–10), with subsequent slower increases (DIV11–12; **Figure S1B**). The secreted BDNF stimulated TrkB which was phosphorylated at Y705 and at Y516 sites (**Figure S1C**). TrkB phosphorylation led to downstream mitogen-activated protein kinase (MAPK) and phospholipase C gamma 1 (PLC γ-1) phosphorylation (**Figure S1C**). These results demonstrate that the neurons transduced with constructs produced and secreted biologically active BDNF.

### In Vivo Experiments

#### Animals

Forty young adult male Wistar rats weighing 250–400 g at the end of the experiment were used in this study (**Table 1**). The animals were bred in the animal facility of the Nencki Institute. Rats were given free access to water and pellet food and were housed under standard humidity and temperature conditions on a 12 h light/dark cycle. Experimental protocols, involving animals, their surgery and care were approved by the First Local Ethics Committee in Warsaw (agreement no 707/2006), in compliance with the European Union animal care guidelines (European Community Council Directive 86/609/EEC). Two groups of rats: lesioned, with no intraspinal injection (N = 4), and non-lesioned, intact (N = 4) were used for HPLC analysis of amino acids. Three groups of rats were behaviorally assessed and tested for mRNA (RT-PCR) and protein (ELISA, Western blot or immunohistochemistry) expression in the spinal cord. These belonged to the following experimental groups: 1) lesioned with intraspinal injection of phosphate buffered saline, pH 7.4 (SP-PBS; N = 8); 2) lesioned with intraspinal injection of AAV-BDNF (SP-BDNF; N = 11); 3) non-lesioned, intact (N = 9). Two SP-BDNF rats died on the 33^rd^ day of the experiment (one of them had an injury of scrotum; autopsy made by histopathologist did not reveal reason of their death), therefore only 9 SP-BDNF animals were used for analysis of biochemical (N = 4) and immunohistochemical (N = 5) parameters. The sixth group, lesioned with intraspinal injection of AAV-EGFP (SP-EGFP; N = 4) was used for immunohistochemistry only.

**Table 1:** Summary of the status of animals and tissue analysis.

Group name (total numberof animals)	Description	Number of animals in analysis		
		Biochemistry(& behavior)	IHC(& behavior)	HPLC
Intact (N = 13)	non-operated	5 (3)	4	4
Spinal (N = 4)	transected	–	–	4
SP-PBS (N = 8)	transected and bilaterally injected with PBS	3 (3)	5	–
SP-EGFP (N = 4)	transected and bilaterally injected with AAV1/2-SYN-EGFP	–	4	–
SP-BDNF (N = 11*)	transected and bilaterally injected with AAV1/2-SYN-BDNF	4 (4)	5 (7*)	–

*Two animals from this group had died before the end of the experiment and thus were tested only behaviorally in the early post-operative period (see text for details).

Numbers in brackets mark those animals which were used for both behavioral and biochemical analyses.

#### Surgical procedure – complete spinal cord transection and intraspinal injections

Surgical procedures were performed as described [28] except that the animals were given a subcutaneous injection of butorphanol analgesic (Butomidor, Richter Pharma, Wels, Austria; 3.3 mg/kg) as a premedication and then anesthetized with Isoflurane (Baxter, Lessines, Belgium, 1–2.5% in oxygen) via facemask. The first laminectomy was performed at the thoracic (Th) 9–10 vertebrae, and the cord was completely transected at the Th9–10 (groups operated for HPLC experiment) or at Th 11–12 (groups operated for the immunohistochemical and RT-PCR/protein assays). The second laminectomy was performed at the Th 11–12 vertebrae to expose the spinal cord for injections. After opening the *dura* and *pia maters* AAV-EGFP, AAV-BDNF or PBS were injected via a fine glass capillary, inserted into the spinal cord about 0.7 mm laterally from the midline, 1 mm in depth. Surgical stereomicroscope Nikon SMZ 1000 was used to control positioning and movement of the capillary and avoid bending of the spinal cord. One µL of either a viral particle solution (3×10^8^ AAV particles) or PBS were injected at a speed of 0.1 µL per minute (sp 101i syringe pump, WPI, Sarasota, FL, USA). Single injections were given bilaterally within half an hour after spinal cord transection. Five minutes after injection, the capillary was removed, the cut tissues were sutured, the skin over the wound was closed with sterile stainless steel staples, and 5 mL of 0.9% NaCl was injected SC. The anti-inflammatory/analgesic Tolfedine (3 mg/kg, SC) and the antibiotic enrofloxacin (Baytril, 2.5%; 0.2 mL/kg, SC) were administered at the end of the surgery, and then daily for 3 and 5 days. The rats were inspected daily and bladders were voided manually. Animals were weighed every 5 days. During the first week after injury, rats lost 10–15% of their preoperative body weight, but most of them gradually regained their preoperative weight at about 4 weeks after injury.

#### Kinematic analysis of treadmill locomotion

Rats were accustomed to the treadmill walking as described previously [28]. After a one-week recovery period following spinal cord transection, locomotion of the rats was examined with the forelimbs and rostral trunk of the animal placed on a platform located 1 cm above the belt, while the hindlimbs were placed on the running treadmill. Rats walked assisted by the experimenter, who secured the proper position of the trunk on the platform to prevent lateral and upward movements of the rostral trunk lying on the platform while the rat was walking on its hindlimbs. One hand of the experimenter surrounded (from the top and lateral sides) the rostral trunk. The experimenter did not raise the rostral trunk up and limited the rostral trunk to be raised up. In order to prevent any training effect suspected to promote locomotor recovery, treadmill locomotor tests occurred only twice during the experiment, between 6 and 17^th^ days (early postoperative period) and between 35 and 52 days (late postoperative period). Testing days of each animal are specified in **Table 2**. The animals were filmed with a digital camera (Panasonic NV-GS400) at 25 frames/s while walking on the moving treadmill. For kinematic gait analysis, equally sized black markers were glued to the shaved skin overlying the femur and tibia heads, tibiofibular articulation, distal metatarsus and distal phalanx of the third toe. The angular excursions in the joints of the hindlimbs were measured during quadrupedal walking in the intact rats at a treadmill speed of 0.05 m/s and compared with those of the spinal rats at the same treadmill speed. The camera was positioned perpendicularly to the longitudinal axis of the animal’s body. Videographic analysis was performed by means of Image–Pro Plus (Media Cybernetics, Silver Spring, MD, USA) software developed to create stick figures of the hindlimb movements with a time resolution twice as fast as that of the camera (50 images/s).

**Table 2:** Locomotor capabilities of spinal rats in PBS- (SP-PBS) and BDNF-treated (SP-BDNF) groups evaluated during hindlimb walking on a moving treadmill.

Group Rat No	Evaluation of motor performance of spinal rats walking on the moving treadmill (modified BBB scale)										Sacrifice day
	Early post-operative period					Late post-operative period					
	No tail stimulation		Tail stimulation		Testing day	No tail stimulation		Tail stimulation		Testing day	
	Level	Score	Level	Score		Level	Score	Level	Score		
SP-PBS											
5.1	1	0	2	4	7	2	2	2	13	47	49
5.2	1	0	2	5	6	2	3	2	13	52	54
5.4	NA	NA	NA	NA	–	2	2	2	13	35	38
Mean ± SD		0±0.00		4.5±0.71			2.3±0.58		13±0.00		
Median		0		4.5			2		13		
SP-BDNF											
4.1	4	11	4	17	16	4	11	4	17	39	39
4.2	4	13	4	17	17	–	–	–	–	–	–
4.3	4	11	4	19	15	4	16	4	17	37	40
4.4	4	11	4	17	14	2	3	2	16	40	40
4.5	4	17	4	17	13	4	11	4	11	39	40
4.6	4	17	4	19	13	4	19	4	17	40	44
4.7	2	3	4	11	13	4	16	4	11	40	41
4.8	2	3	4	13	11	4	18	4	12	42	44
4.9	4	15	4	17	10	–	–	–	–	–	–
4.10	4	17	4	19	9	4	11	4	17	37	47
4.11	1	0	2	3	8	4	18	4	15	47	49
Mean ± SD		10.7±6.13		15.4±4.80			13.7±5.14		14.8±2.68		
Median		11		17			16		16		

Differences between scores achieved with- and without tail stimulation in SP-BDNF group in early post-operative period are significant (Wilcoxon test, P = 0.005). Differences between SP-PBS and SP-BDNF groups without tail stimulation in late post-operative period are also significant (Mann-Whitney U test; P = 0.02). NA – not analyzed.

BBB scale modified by Antri and coworkers [54], [55] was used for assessment of bipedal treadmill locomotion in adult, spinalized rats. There are four major levels and 22 scores of recovery of motor capabilities, where level 4 and score 22 correspond to locomotion of intact animal. Animals were tested twice during the experiment, in the early and late post-operative period (exact testing days are shown).

#### Evaluation of treadmill locomotion by means of the modified BBB scale

The locomotor capabilities of the animals were also evaluated using the modified Basso-Beattie-Bresnahan (mBBB) scale for assessing the movement of the hindlimbs with the forelimbs placed on a platform [54], [55]. The scale is divided into four levels of the major steps of recovery and 22 scores of motor capabilities. Level 1 (scores 0–1), level 2 (scores 2–9), level 3 (score 10), level 4 (scores 11–22). Level of 4 and a score of 22 on the mBBB scale have been ascribed to the locomotion of intact rats. Thirty step cycles per animal where evaluated by two independent observers analyzing video recorded treadmill locomotion. Weight support was defined as an elevation of the hindquarter [56].

#### Tissue dissection for ELISA, Western blot and real-time quantitative RT-PCR analysis

Rats were deeply anesthetized with a lethal dose of pentobarbital (80 mg/kg body weight, i.p.) and perfused transcardially with ice-cold saline. The vertebral column was excised, placed on ice, and spinal cords were removed in a cold room and frozen on dry ice. Slices of 0.8 mm thickness were then cut on a McIlwain tissue chopper (Ted Pella Inc., Redding, CA, USA) and split into left (L) and right (R) portions to evaluate potential differences in the impact of lesion and injections between hemi-cords. Every second slice of a hemicord was subjected to ELISA and Western blot or to quantitative RT-PCR (qPCR). For each animal, slices from segments Th10–11, Th11–12 (lesion site), L1–2 (injection site) and L3–6 were pooled and stored at −80°C.

#### Preparation of homogenates for ELISA and Western blot analysis

Crude tissue homogenates (20% w/v) were prepared in 100 mM Tris buffer (pH 7.0) containing 5% glycerol, 0.1% SDS, Complete Protease Inhibitor Cocktail and phosphatase inhibitors (P2850, P5726, Sigma-Aldrich), with the addition of 200 µM phenylmethyl-sulphonyl fluoride (PMSF; Sigma-Aldrich) and 157 µg/mL benzamidine hydrochloride (Serva, Heidelberg, Germany). A glass/Teflon Potter Elvehjem tissue grinder or glass/glass grinder were used. Homogenates were divided into two portions: the one for the BDNF ELISA was supplemented with 2% BSA, 1M NaCl and 2% Triton X-100. The supplemented and non-supplemented portions were incubated on ice for 30–60 minutes and centrifuged at 11 600×*g* for 30 min at 4°C. Immediately afterwards BDNF ELISA was performed following the manufacturer’s instructions (Millipore, Billerica, MA, USA). The supernatants from the non-supplemented extracts were used for Western blotting analysis (BDNF, KCC2) and for ELISA (GABA) (Labor Diagnostika Nord, Nordhorn, Germany). All samples were run in duplicate.

### Western Blot Analysis

The culture medium was boiled for 5 min at 98°C in sample buffer (62.5 mM Tris (pH 6.8), 2% SDS, 10% glycerol, 0.01% bromophenol blue, and 100 mM DTT or 2% β-mercaptoethanol). *Cells* were collected in Tris-based SDS lysis buffer (50 mM Tris (pH 8.0), 0.5% SDS, 1 mM DTT, and 1X Complete Protease Inhibitor Coctail (Roche Applied Science, Indianapolis, IN, USA)). NP40 (1%) and 1X PhosStop Phosphatase Inhibitor Cocktail (Roche Applied Science) were added to the lysis buffer when protein phosphorylation was to be analyzed. After sonication, homogenates were centrifuged (30 min, 11 600×g, 4^o^C) and supernatants were collected. Protein was determined with the BCA Protein Assay (Thermo Fisher Scientific, Waltham, MA, USA), The total protein in the *tissue samples* (preparation of homogenates described above) was measured in non-supplemented s1 supernates with Bradford method [57]. 30–20 µg total protein was subjected to SDS-PAGE. After protein transfer to PVDF membranes (BioRad), membranes were blocked for 1 h at RT with 5% BSA and incubated with primary antibody diluted in blocking solution overnight (4°C). Primary antibodies were then detected with HRP-conjugated secondary antibodies with ECL reagent (GE Healthcare) as substrate. Blots were reprobed without stripping with antibodies to β-tubulin or β-actin for loading control.

### Analysis of BDNF, VGluT1, VGluT2, GAD67, GAD65 and KCC2 by Quantitative RT-PCR

Total RNA was isolated using ZR RNA MiniPre kit (Zymo Research Corporation, Irvine, CA, USA), followed by the DNaseI treatment, as described earlier [58]. Briefly, total RNA (0.5 - 1 µg) was converted into cDNA using Transcriptor High Fidelity cDNA Synthesis Kit reagents with random hexamers as primers (Roche Applied Science). Quantitative PCR for BDNF, VGluT1, VGluT2, GAD67, GAD65, and KCC2 was performed by means of TaqMan probes using the LightCycler 480 sequence detection system (Roche Applied Science, Indianapolis, IN, USA). Dual color qPCR was performed for each transcript in parallel with a probe specific for glyceraldehyde-3-phosphate dehydrogenase (GAPDH) (Universal Probe Library Rat GAPD Gene Assay, Roche Applied Science). Gene-specific probes, forward and reverse primers as designed by Universal ProbeLibrary Assay Design Center were used. For the amplicons’ sequences see **Table S1**.

### Tissue Extraction and Sample Preparation for HPLC

Tissue extraction and HPLC were performed as described by Skup and co-authors [47]. Briefly, 5 weeks after transection, the rats were decapitated, immediately after that the corps were cooled with dry ice, and spinal cords were removed from the vertebral columns and divided into mid- and caudal thoracic (Th), rostral lumbar (L1–2) and caudal lumbar (L3–5) segments. Samples were weighed and frozen (−70°C) until HPLC analysis. Immediately prior to HPLC, tissue samples were sonicated in 0.1 M HClO_4_ solution containing 0.4 mM Na metabisulfite and centrifuged at 7000×*g* for 5 min at RT. Supernatants were filtered (Spartan 3/0.2 PA nylon syringe filter, Schleicher & Schuell) and kept on ice until injection into the HPLC system. Simultaneous measurements of glutamate (Glu), aspartate (Asp), glycine (Gly) and gamma-aminobutyric acid (GABA), were performed using the Merck-Hitachi HPLC system with a fluorescence detector (F 1050). Prior to injection into the apparatus, samples (20 µl) were automatically derivatized with orto-phtaldialdehyde (Merck, Darmstadt, Germany) and β-mercaptoethanol (Sigma-Aldrich) in 0.5 M borate buffer with methanol (1∶9) and injected with an autosampler (Merck-Hitachi, LaChrom, L-7250). The separation was performed using a Li Chromspher 18 RP 250×4.6×5 column and a mobile phase that was a binary eluent of 50 mM CH_3_COONa (pH 7.0) and methanol under gradient conditions (CH_3_OH from 26% to 40% during 0.5 h). The column temperature was maintained at 35°C. The flow rate was 1.0 mL/min. All measurements were performed in quadruplicate. The data were collected and processed using Chromatography Data Station Software (version 3.1.1, Merck-Hitachi Model D-7000).

### Immunohistochemistry

Rats were deeply anesthetized with a lethal dose of pentobarbital (80 mg/kg, i.p.) and transcardially perfused with 0.01 M PBS followed by 4% paraformaldehyde in 0.1M PBS for 20 min. The spinal cords were removed, left in fixative for 24 hours at 4°C, cryoprotected in 30% sucrose in 0.1 M PBS and stored at 4°C. Frozen segments were sectioned (25 µm) in the sagittal plane on a Leica cryostat. Prior to immunostaining, sections were incubated in 0.01 M PBS with 0.2% Triton X-100 (PBS-T) and blocked with 5% normal goat serum for 1 hour at RT. Following an overnight incubation at 4°C with primary antibodies, sections were washed 3 times with PBS-T (3–5 min) and incubated with DyLight 488 goat anti-rabbit (1∶500), DyLight 594 goat anti-mouse (1∶500; Jackson Immuno Research, Suffolk, UK) or Alexa Fluor 488 goat anti-rabbit (1∶500) and Alexa Fluor 594 goat anti-mouse (1∶500, Alexa Fluor, Life Technologies Corporation (Molecular Probes), Carlsbad, CA, USA) antibodies for 45 min at RT. The reaction was terminated by washes (3 times with PBS-T; 3–5 min), followed by 0.01 M PBS. Next, sections were immersed for 5 min in 0.2 µM bisbenzimide H 33258 (Hoechst; Sigma-Aldrich) to stain nuclei, mounted in Mowiol (Mowiol 4–88, Sigma-Aldrich (Fluka), St. Louis, MO, USA), coverslipped, and kept in the dark at 4°C until analysis.

### Evaluation of Completeness of Transection

Spinal cord injury was classified as complete based on: (1) a functional basis as judged by evaluation of motor capabilities; (2) postmortem histological inspection of the dissected spinal cords under the microscope. Moreover, (3) immunolabeling for serotoninergic (5HT) axons was done on the sagittal, serial sections from the spinal cords processed for immunohistochemistry, and (4) 5-HT was measured in a group of animals which was used for HPLC analysis. This examination showed: (a) a good reproducibility of the lesion location and its size (**Figure S2**); (b) no 5HT-positive cells or fibers below the lesion site (**Figure S3**), and (c) 5HT concentration decrease to less than 2% of control values in the lumbar segments (intact controls: L1∶1.55+/−0.491; L3∶1.81+/−0.299; Spinalized: L1∶0.03+/−0.018; L3∶0.03+/−0.090 pmol/mg wt). See also [59]. Since the source of residual 5HT may be spinal neurons [60] we take all four measures as a strong indication of the lesion completeness.

### Microscopy and Image Analysis

Sections were examined using a Nikon Eclipse 80i microscope equipped with 10x (0.30 N.A.), 20x (0.50 N.A.) and 40x (0.75 N.A.) objectives. Digital images were captured with a monochromatic CCD camera model Evolution VF (Media Cybernetics, Inc. Silver Spring, USA). An Image-Pro Plus 5.0 digitizer was used for the tiling, editing, assembling and image analysis of the digital microphotographs. The sections processed for cMYC and KCC2 IF were examined with confocal microscopy (confocal inverted microscope Leica DM IRE 2). Confocal z-stacks of optical sections of a thickness 0.16 µm were obtained using a HCX PL APO 63x oil-immersion objective lens. Photographs were assembled using Adobe Photoshop software. Drawings and graphs were edited in Corel Draw 15.

### Statistical Analysis

The homogeneity of variance for each variable in the tested groups was verified using the Levene’s test. If an assumption of the homogeneity of variances was verified, the two-way ANOVA and the Tukey *post-hoc* tests were used to compare the vast majority of biochemical data. Non-parametric statistics: Mann-Whitney U test for comparisons between independent samples and Wilcoxon test for the related samples were used in cases with unequal variances. Correlation analysis of biochemical parameters was done using Spearman correlation test. *Statistica* software (StatSoft Inc, Tulsa, OK, USA) was used here.

## Results

### Assessment of AAV-1/2 Transduction in the Transected Adult Rat Spinal Cord

**Figure 1:**
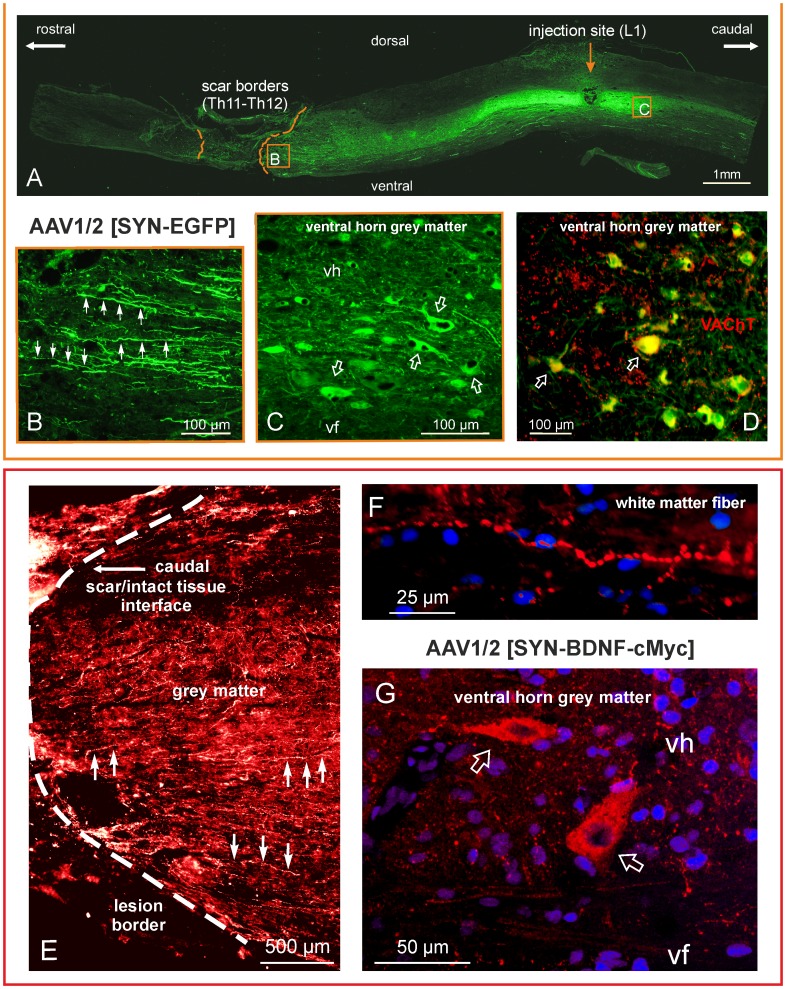
Spinal cord transduction with an AAV1/2 vector expressing EGFP or BDNF with cMYC tag at 7 weeks after spinal cord transection. Upper panel: (A) Tiled images taken of the thoraco-lumbar segments of a spinal cord from a spinalized rat that received the EGFP transgene; micrographs were taken on a fluorescence microscope at ×10 magnification. The dashed lines delineate the edges of the scar. (**B**) An enlarged micrograph of the framed area in (**A**) documenting the numerous EGFP-positive fibers (arrows) running along the grey matter in close proximity to the lesion site. (**C**) An enlarged micrograph of the framed area in (**A**) showing transduced cells of neuronal morphology in the ventral horn (arrows). (**D**) A merging of the EGFP expression signal (green) with vesicular acetylcholine transporter (VAChT, red) immunolabeling shows their colocalization (yellow), which confirms that the transduced cells are motoneurons (arrows). Bottom panel: The spinal cord from a rat that received the BDNF-cMYC transgene. (**E**) cMYC immunostaining detected BDNF-cMYC-positive neuronal fibers (exemplified by arrows) below the transection, in the lower thoracic segments of the spinal cord. Fibers approach and encroach on the scar from its caudal aspect. The white dashed line delineates the caudal border of the scar. A dense mesh of small caliber BDNF-cMYC-positive fibers prevails in the grey matter (**E**) whereas large caliber varicose fibers appear in the white matter (**F**). (**G**) The confocal microscope microphotograph shows two BDNF-cMYC-positive, large size neurons (arrows) of the L2 ventral horn. Hoechst nuclear labeling is shown in blue. Abbreviations: vh – ventral horn, vf – ventral funiculus.

We determined immunohistochemically transduction 7 weeks post-lesion and bilateral AAV-EGFP (N = 4) or AAV-BDNF (N = 5) injection. The EGFP protein and the cMYC-tag of the BDNF transgene were markedly expressed below the transection site, in neurons of all spinal laminae (**Figure** **1** **, see also** **Figure 6E–J**). EGFP-expressing cells were dispersed over a range of 8 mm rostro-caudally from the injection site with some of them sending fibers penetrating the scar (**Figure 1A–C**). Single EGFP-expressing neurons were found up to 10 mm away from the injection site. Some of them were α-motoneurons, as concluded from their cholinergic phenotype and presence of C-terminals [61] apposing EGFP-expressing motoneuronal perikarya (**Figure** **1D**). Immunohistochemistry for cMYC-tagged BDNF revealed that recombinant BDNF was strongly expressed in neurons of the I and III laminae of the dorsal horn, in the intermediate zone and in ventral grey large neurons of motoneuron size (**Figure 1G** and **6E–J**). Longitudinal c-MYC-labeled fibers were observed running toward the lesion border in both grey and white matter, indicating that overexpressed BDNF is transported along neuronal processes and may be widely available for the neuronal network caudal to the transection site (**Figure 1E, F** and **6E, F**).

**Figure 2:**
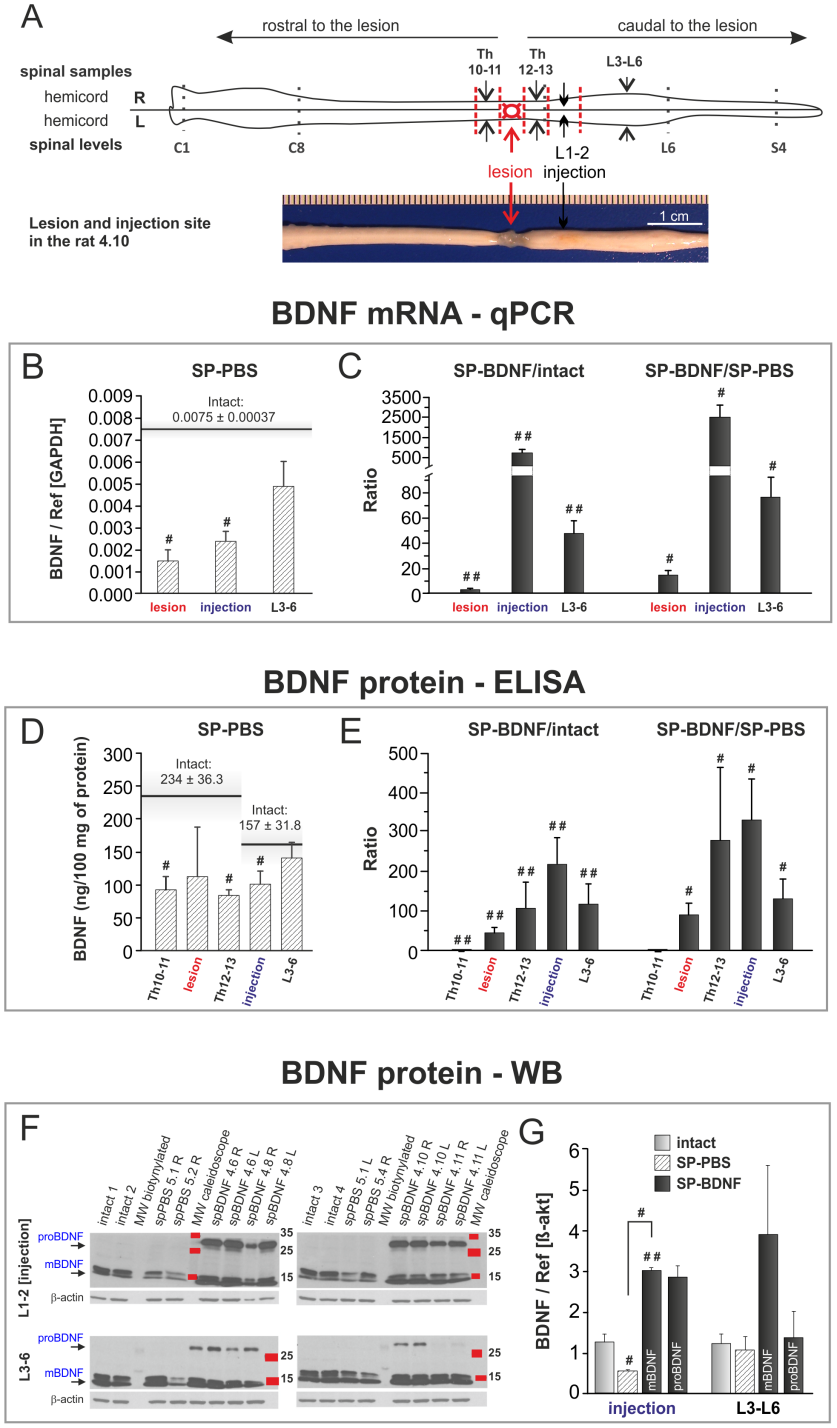
AAV-BDNF counteracts thoraco-lumbar BDNF deficits and causes BDNF overproduction in spinal segments 7 weeks after spinal cord transection. (**A**) Diagrammatic representation of spinal cord microdissection for biochemical analyses. A photograph exemplifying the lesion and injection site is shown below. AAV-BDNF was injected separately to each hemicord, and injection efficacy was analyzed for samples from right (R) and left (L) hemicords, except for the lesioned Th11–12 segment. Afterwards, the means from L and R hemicords were calculated and presented in the **B–G**. BDNF mRNA levels were evaluated with qPCR (**B, C**). BDNF concentration was measured with ELISA in the s1 fraction obtained from the homogenates of spinal Th10-L6 segments (**D, E**), and changes in BDNF mature (mBDNF) and precursor (proBDNF) forms were evaluated by Western blot analysis (**F, G**). Spinal cord transection leads to a decrease in the BDNF mRNA level (**B**; hatched bars) and protein concentration (**D**; hatched bars) in the lesion site, low thoracic and rostral lumbar segments. Black horizontal lines in **B** and **D** mark the control values for the intact animals. AAV-BDNF causes significant increase of the level of BDNF transcript (**C**; black bars) and BDNF concentration (**E**; black bars) in the transection site and in the spinal segments caudally to the transection. Bars in **C** and **E** show the ratios of the means of BDNF mRNA (**C**) and protein (**E**) concentration in spinal BDNF-treated rats (SP-BDNF) to that in the intact animals (left panels) and in SP-PBS rats (right panels). (**F**) Representative Western blots show the occurrence of mBDNF in individual intact, SP-PBS and SP-BDNF rats and indicate, that proBDNF is clearly detectable in SP-BDNF rats; in the intact and SP-PBS rats proBDNF is below the level of detection. (**G**) Relative optical density of mBDNF bands in respective groups indicate that in SP-BDNF rats mBDNF is elevated above controls in the rostral lumbar segment and tends to increase in the caudal lumbar segments (P = 0.061); 2 to 4 Western blot performed for each sample were analyzed and data were normalized to β-actin. Bars represent means±SD (**B–E**) or ± SEM (**G**) from 5 intact, 3 SP-PBS and 4 SP-BDNF rats. Mann-Whitney U test, ^#^P<0.05, ^##^P<0.01.

**Figure 3:**
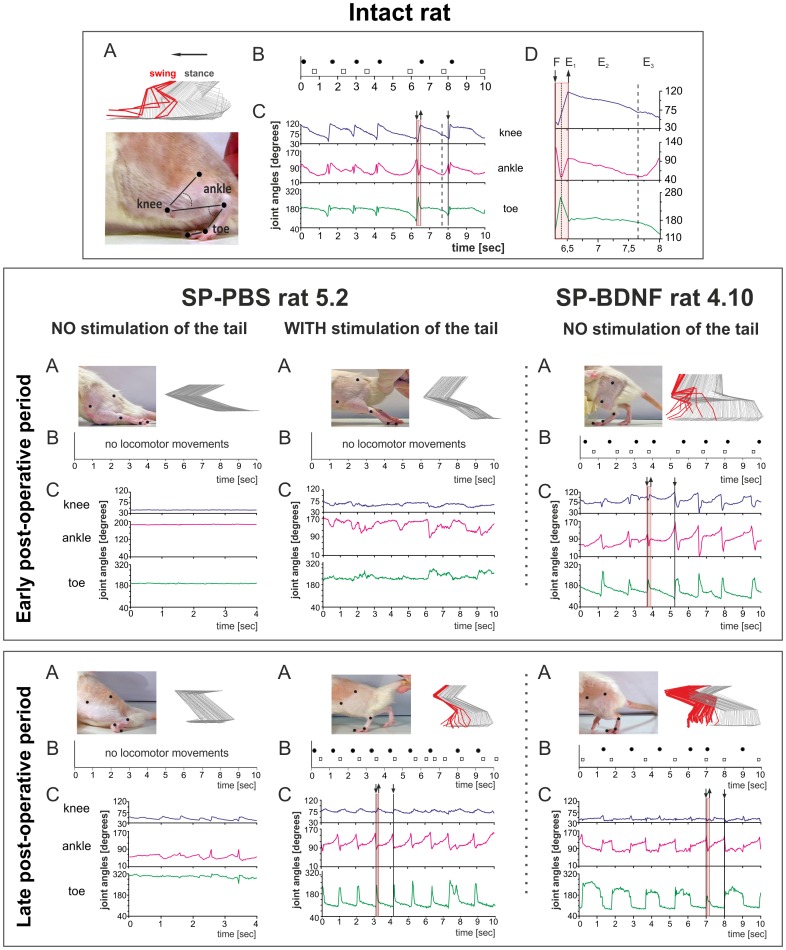
BDNF overexpression leads to an early recovery in locomotor function. Comparison of the treadmill locomotion of the intact, SP-PBS and SP-BDNF rats. Upper panel: kinematic analysis of the gait of an intact rat during locomotion on the moving treadmill (**A**) Stick figure showing the angular excursions of the knee, ankle and toe joints during one step cycle during slow (0.05 m/s) treadmill locomotion in an intact rat. To measure the angular excursion of the hindlimb joints, the black markers were glued to the shaved skin overlying the femur and tibia heads, tibiofibular articulation, distal metatarsus and distal phalanx of the third toe (bottom, **A**). A digital Panasonic camera (NV-GS400 3CCD) was used to capture video images of the hindlimbs during treadmill locomotion. Software based on Image–Pro Plus was used to create the stick figures of the hindlimb movements with a resolution twice as fast as that of the camera (i.e., 50 images/s). Every stick image was artificially separated from the next by the same coefficient to avoid superimposing neighboring stick images. (**B**) The footprints of both hindlimbs of an intact rat corresponding to the beginning of the foot contact with the treadmill during locomotion as taken from the video. Black dots - left leg; squares - right leg. (**C**) Angular excursions in the knee, ankle and toe joints during 10 s of treadmill locomotion. Downward deflection of the angular traces indicates flexion movement. (**D**) A framed plot from **C** of the angular excursions during one step was enlarged to indicate the phases of locomotion (F-E1 correspond to the swing and E2–E3 to the stance phase). Middle panel*:* examples of treadmill locomotion during the early post-operative period (the second week after surgery) of the spinal rats injected with PBS or with AAV-BDNF. None of the SP-PBS rats were able to perform locomotor movements when their hindlimbs were placed on the moving treadmill (exemplified in the left column; rat 5.2). Addition of tactile stimulation of the tail produced some agitation in both hindlimbs but did not evoke locomotor movements in the PBS-treated rats (central). Contrary to SP-PBS treated rats, no tail stimulation had to be used to trigger locomotion with body weight support in SP-BDNF rats. Periods of alternating, treadmill walking with body weight support and plantar foot placement but reduced rhythmicity were observed in eight out of the eleven rats overexpressing BDNF (exemplified in the right column, rat 4.10). Bottom panel: examples of treadmill locomotion of the spinal rats in the late (about 40 days) postoperative period. None of the SP-PBS rats could support their body weight or perform plantar foot placement (left); tail stimulation triggered locomotor movement in the PBS-treated rats (central). The locomotor capabilities of the hindlimbs in the SP-BDNF group improved profoundly in rats that were previously classified at the lowest level of the mBBB scale, whereas worsened in rats that walked well in the early post-surgery period (exemplified in the right column, rat 4.10). In that group, stimulation of the tail attenuated the quality of locomotion in rats that walked well without tail stimulation (**Table 2**, rats with scores 16–19). Weight support is defined as an elevation of the hindquarter [56].

**Figure 4:**
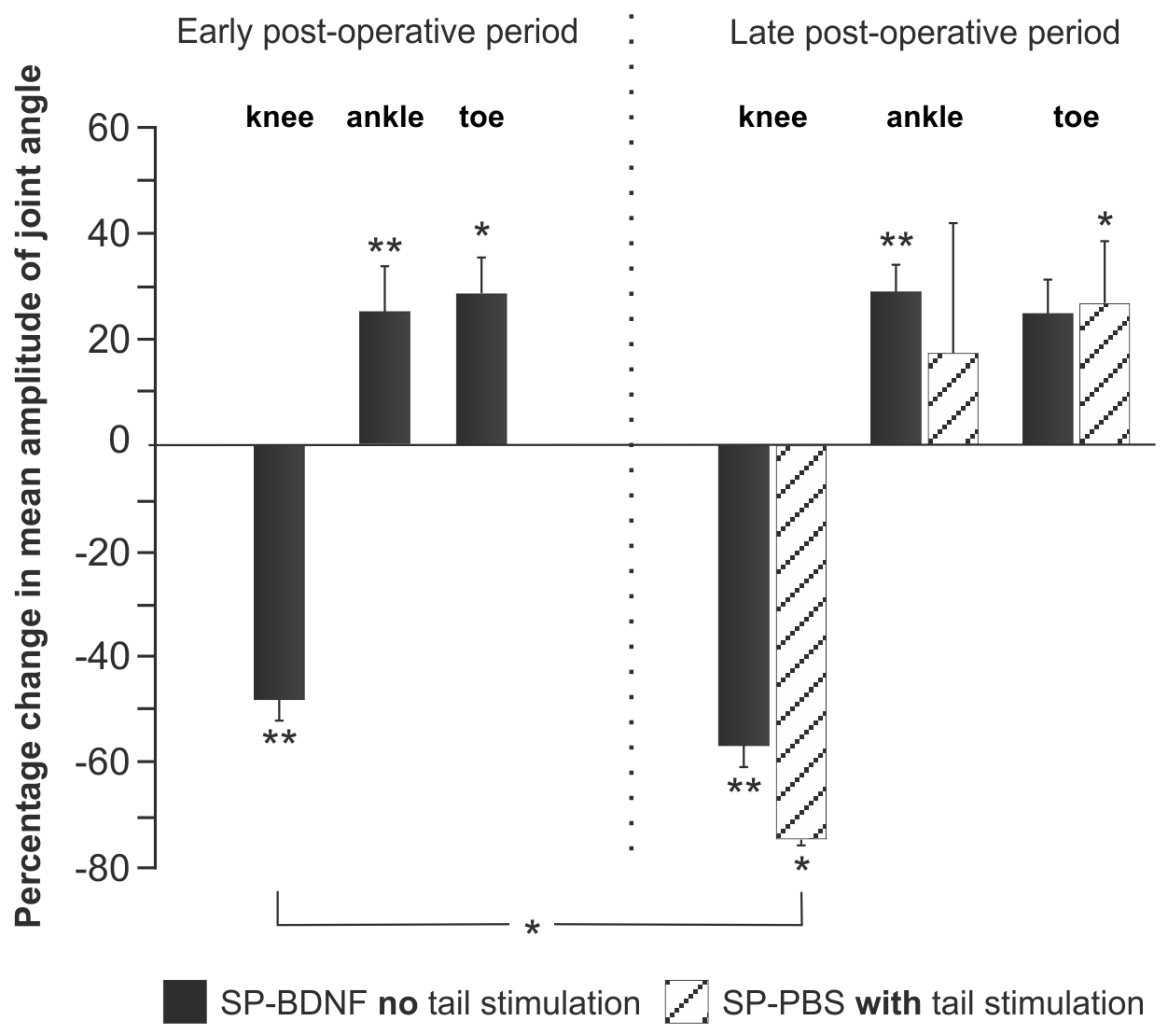
Amplitude of movements in the knee, ankle and toe joints during treadmill locomotion. The angular excursions in spinal rats expressed as a percentage change in intact animals during the early and late postoperative periods are shown. At early period SP-PBS did not walk on the moving treadmill even when the tail stimulation was added therefore no percentage change is shown. Black bars correspond to angular excursions of SP-BDNF rats walking without tail stimulation. Hatched bars indicate angular excursions during treadmill locomotion in SP-PBS rats triggered by stimulation of the tail. In all animals the angular excursions were measured in 10 consecutive step cycles, performed at the same speed of the treadmill (0.05 m/s). Bars represent means ± SEM from 3 SP-PBS and 11 SP-BDNF rats (early period) and 3 SP-PBS and 9 SP-BDNF rats (late period). Asterisks above the bars indicate significant differences between spinalized rats and intact controls; an asterisk above the square brackets indicates significant differences between the SP-PBS and SP-BDNF groups: Mann-Whitney U test, ^#^ P<0.05,^ ##^ P<0.02.

**Figure 5:**
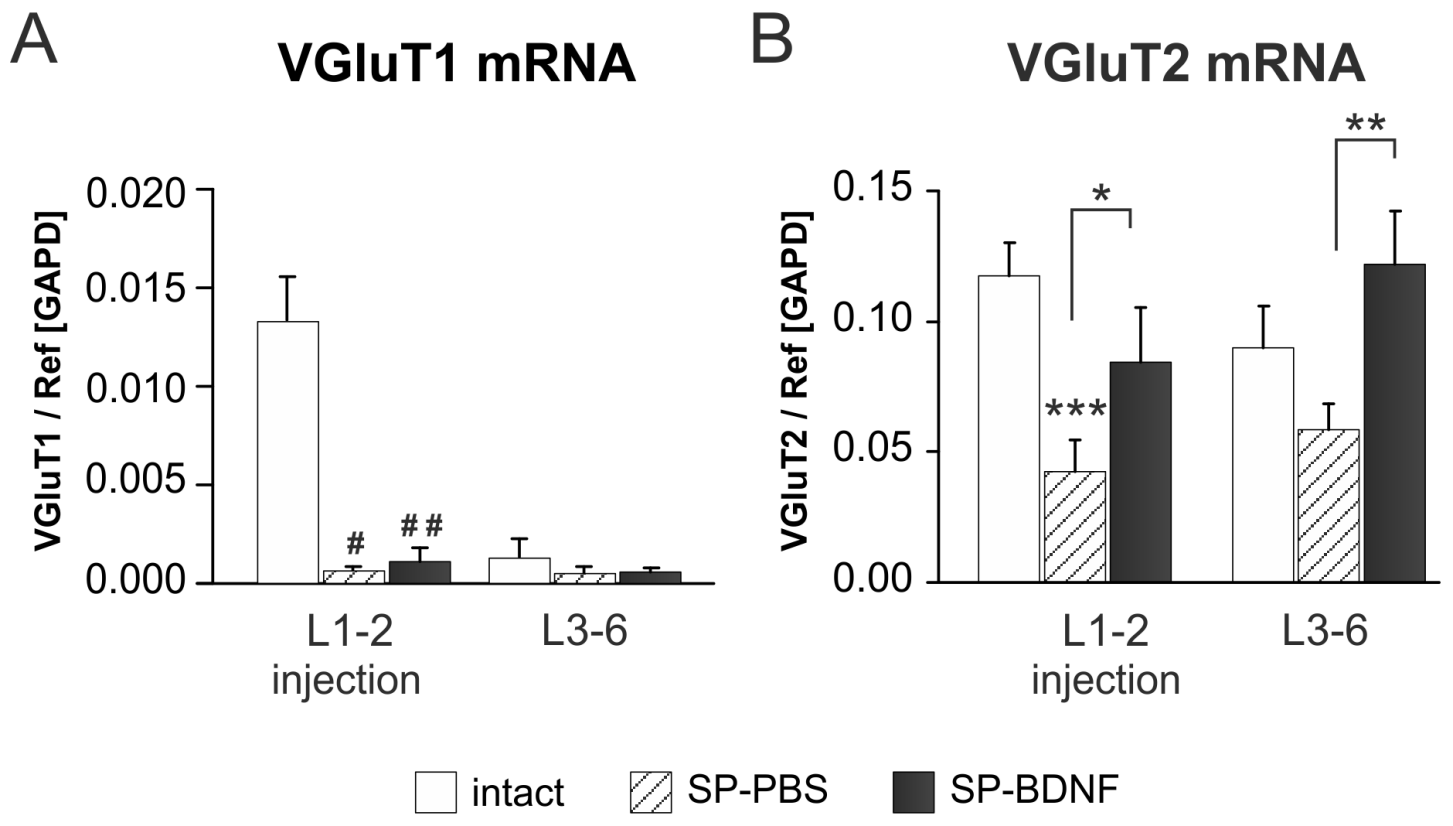
The effects of spinal cord transection and BDNF overexpression on segmental vesicular glutamate transporter 1 (VGluT1) and 2 (VGluT2) transcripts level. (**A**) Spinal cord transection leads to a significant decrease in VGluT1 mRNA level in L1–2 segments and to less pronounced decrease in L3–6 segments (hatched bars). In SP-BDNF rats VGluT1 mRNA goes through similar to SP-PBS rats reductions, both in L1–2 and in L3–6 segments (black bars). (**B**) Spinal cord transection causes a significant decrease in VGluT2 mRNA levels in rostral and a tendency to decrease in caudal spinal cord segments (hatched bars). In SP-BDNF rats VGluT2 transcript level is significantly higher than in SP-PBS rats both in L1–2 and L3–6 segments, where it tends to be higher than in control rats (black bars). Data are the means ± SD from 5 intact, 3 SP-PBS and 4 SP-BDNF rats. Mann-Whitney U test was used to compare VGluT1 mRNA values: # P<0.05, ## P<0.02; Two-way ANOVA with Tukey post-hoc tests were used to compare VGluT2 mRNA values: *P<0.05, ***P<0.001. Asterisks above the bars indicate significant differences between spinalized rats and intact controls; asterisks put above the square brackets indicate significant differences between the SP-PBS and SP-BDNF groups.

**Figure 6:**
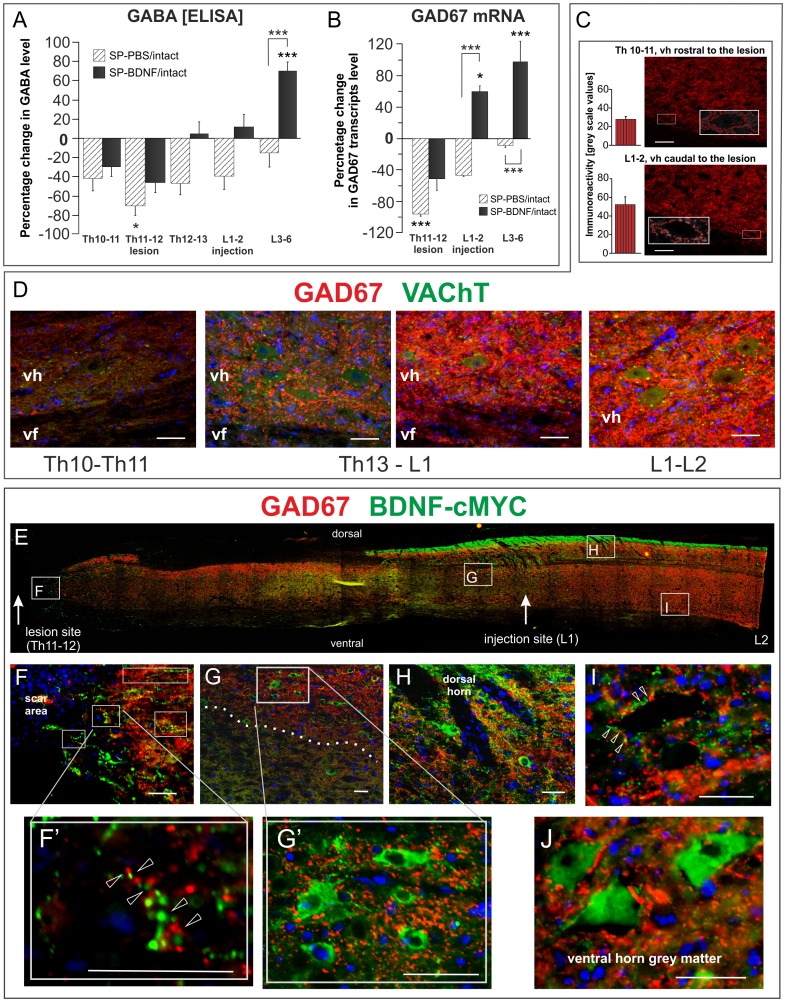
AAV-BDNF-induced segmental changes of GABA and GAD67 mRNA 7 weeks after spinal cord transection. (**A, B**) BDNF overexpression leads to an increase of GABA and GAD67 mRNA levels exceeding control levels in L3–6 segments. Hatched and black bars represent their segmental levels in SP-PBS and SP-BDNF rats, respectively, expressed as a percentage of the level in intact animals. GABA concentration in intact rats equals to 2.38±0.16 µmol/100mg of protein. Asterisks above the bars indicate significant differences between spinalized rats and intact controls; asterisks above the square brackets indicate significant differences between the SP-PBS and SP-BDNF groups. Data are the means ± SEM from 5 intact, 3 SP-PBS and 4 SP-BDNF rats. Two-way ANOVA with Tukey *post-hoc* tests were used, *P<0.05, ***P<0.001. (**C**) Labeling intensity of GAD67-positive boutons terminating on large neurons of the ventral horn (insets) is lower in Th10–11 than in L1–2 segments (means ± SD measured in 26 and 29 boutons, respectively). An example. (**D**) GAD67-immunolabeling of fibers and boutons (red) terminating on motoneurons (immunolabeled for VAChT, green) in the longitudinal parasagittal section of the spinal cord of the rat that received BDNF transgene with cMYC tag. Note a gradient of GAD67 immunolabeling intensity, which is lower in the thoracic region above the transection site (Th10–11, devoid of BDNF-cMYC expression - left), than in the lumbar (L1–2) region, enriched in BDNF-cMYC. Abbreviations: vh – ventral horn, vf – ventral funiculus. Bars equal to 50 µm. (**E**) A reconstruction from fused microphotographs of a thoraco-lumbar longitudinal parasagittal section of the spinal cord from the SP-BDNF rat shows widespread distribution of BDNF-cMYC immunostaining (green) caudally to the lesion. Framed areas on **E** (showed in higher magnification in **F–I)** demonstrate that BDNF transgen expression spatially correlates with GAD67 labeling (red). BDNF-cMYC is present in fibers (arrowheads), some with varicosities (**F, F’, H, I**) and in neuronal perikarya throughout grey matter (**G, G’, H, J**). Except for the scar area devoid of GAD67 immunolabeling (**F**), in other regions BDNF-cMYC signal is associated with intense GAD67 immunoreactivity detected in cell perikarya and fibers (the area above the dashed line in **G** and the dorsal horn region in **H**). No co-localization of the two markers was observed; arrowheads indicate separate cMYC and GAD67 signals in fibers (**F’**) including boutons apposing large BDNF-cMYC negative neurons (**I**). BDNF-cMYC positive neurons are GAD67 negative and receive no inputs from BDNF-cMYC expressing projections (**G, G’, H, J**). Hoechst labeling (in blue) marks cell nuclei. Bars equal to 50 µm.

### BDNF Overexpression Raises BDNF Levels Beyond Normal Levels in the Thoracic and Lumbar Segments Seven Weeks after Transection

We performed qPCR and ELISA to measure BDNF transcript and protein levels after transection in rats without and with transgene overexpression. To evaluate segmental responses of neural circuits located rostral and caudal to the transection, we sampled the spinal cords, as illustrated in **Figure** **2A**. In the intact spinal cord BDNF mRNA levels in the low thoracic segments equaled those in the lumbar segments, whereas BDNF protein concentration in low thoracic segments was 1.5 fold of that in the lumbar segments (Wilcoxon test, P<0.04). Transection led to a significant decrease in BDNF mRNA both in the lesion and injection sites (Mann-Whitney U test, P<0.05), with a tendency to decrease in L3–6 (**Figure** **2B**). BDNF protein levels decreased in the transected segment (by 52%), in neighboring Th regions (a 60% decrease rostral and a 64% decrease caudal to the transection site) and in the L1–2 segments (by 36%) (Mann-Whitney U test, P<0.05 for Th10–11, Th12–13 and L1–2 regions) (**Figure 2D**).

AAV-BDNF injection resulted in a marked upregulation of mRNA BDNF expression levels (Mann-Whitney U test, P<0.02 vs intact). In the rostral lumbar segments, the mRNA BDNF levels were over 500 times above control values (**Figure 2C**). These changes were highly correlated with changes in BDNF protein (r = 0.860; P<0.05), detected by ELISA (**Figure 2E**). The BDNF protein concentration was the highest in the injected L1–2 segments, where it was on average 210 times higher than in intact rats (Mann-Whitney U test, P<0.02) and 320 times higher than in SP-PBS rats (Mann-Whitney U test, P = 0.05); see SP-BDNF/intact and SP-BDNF/SP-PBS ratios shown in **Figure 2E**. For L3–6, the ratio was 130∶1 with respect to both groups (P<0.02 and P = 0.05, respectively). BDNF protein levels detected in the scar formed at the lesion site, and in the low thoracic segments (Th12–13) were also higher than those of controls (P<0.02) (**Figure 2E**). For the raw data see **Figure S4**.

Western blot analysis showed that the level of the mature form of BDNF (mBDNF) in SP-PBS group, in the L1–2 segments, was significantly lower than that in the intact control (Mann-Whitney U test, P<0.05). In SP-BDNF group mBDNF level was, in the L1–2 segments, significantly higher than in the intact controls (P<0.02). The precursor form of BDNF, which produced strongly immunolabeled band in SP-BDNF rats was below the level of detection in samples from the intact and SP-PBS rats. These results confirmed BDNF overexpression caudal to the transection and revealed that both the precursor and mature forms of BDNF were elevated (**Figure 2F–G**).

### BDNF Overexpression Leads to an Early Improvement in Locomotor Function

Both SP-PBS and SP-BDNF rats were paraplegic after complete spinal cord transection at low thoracic segments but their motor abilities were clearly different.

#### SP-PBS group

During the early post-operative period, the hindlimbs of the SP-PBS rats when placed on the moving treadmill showed no locomotor movements and were dragged on the treadmill belt with the feet on the dorsum (**Fig. 3**). The motor capabilities of these rats were ranked at the lowest level (level 1) of the mBBB scale (**Table 2**). Light pressure stimulation of the tail, which is a prerequisite for triggering treadmill locomotion in spinal rats, evoked only weak and abortive hindlimb movements (level 2, scores of 4 and 5; **Table 2** **,** **Figure 3** **, Video S1**). In the late post-operative period the locomotor capabilities of the hindlimbs of the SP-PBS rats did not improve (**Table 2** **,** **Figure 3** **, Video S2**), confirming our previous results [28]. However, when tactile stimulation of the tail was added, it triggered frequent alternating locomotor movements, with steps performed with plantar foot placement and occasional weight support (**Table 2**, **Figure 3** - central part of the bottom panel). These rats reached level 4 on the mBBB scale with scores of 13.

#### SP-BDNF group

In contrast to SP-PBS rats, in the early post-injury period treadmill locomotion of eight out of eleven SP-BDNF rats reached level 4 of the mBBB scale with scores ranging between 11 and 17 (**Table 2**, **Figure 3** **, Video S1**). Importantly, these rats did not require tactile stimulation of the tail to trigger treadmill walking. They frequently raised their hindquarter and performed steps with plantar feet placement, lifting their feet off the treadmill belt. In two of the other three SP-BDNF rats, stimulation of the tail was necessary to trigger alternating movements of both hindlimbs (mBBB scores 11 and 13) whereas the motor capabilities of one SP-BDNF rat was poor, similarly to those of the SP-PBS rats (level 1 on the mBBB scale) (**Table 2**).

In the late post-operative period, out of the six SP-BDNF rats that previously walked receiving high mBBB scores (two other died), two improved, one remained at the same level, whereas the other three worsened (**Table 2**). Generally, stimulation of the tail improved treadmill locomotion of rats receiving low mBBB scores but in rats that were classified at the high level of mBBB scale this additional stimulation worsened their treadmill locomotion (**Table 2**). In three other rats that were classified at the lowest level of the mBBB scale in the early postoperative period, a clear improvement in locomotor capabilities was observed (**Table 2**). They reached level 4 of the mBBB scale, with scores of 16, 16 and 18; no tail stimulation was required to trigger their hindlimb locomotion. On the contrary, their locomotor movements deteriorated when stimulation of the tail was added. In general, an overstimulation of the locomotor network in treadmill walking rats classified at the high level of mBBB scale increased the number of non-alternating steps and triggered air-stepping movements that were superimposed on the already initiated swing movements.

Differences between the treadmill locomotion of SP-BDNF, SP-PBS and the intact rats are illustrated by the stick figures of the hindlimbs and by the footstep patterns (**Figure 3**). Analysis of joint angles (**Figure 4**) did show that in SP-BDNF rats the amplitude of movement in the knee joints of the hindlimbs were attenuated by 48% during the early postoperative phase compared to the intact rats (the Mann-Whitney U test; P = 0.01). Higher amplitudes in the ankle and toe joints (by about 25%; P<0.01 and P<0.05, respectively) appeared to counterbalance smaller movements in the knee joints. This compensatory effect diminished during the late post-operative period in the SP-BDNF rats as the amplitude of movement in the knee joint tended to decrease further (by 9% compared to that in earlier period (n.s.) and by about 57% compared to the intact rats, P = 0.01), but that in the distal joints remained at a level similar to that observed earlier (**Figure 4**). It resulted in reduced possibility of the compensation of the deficit observed in the knee joint which may be achieved by higher amplitude of movements at the distal joints. Together with increased frequency of clonic movements they attenuated the quality of locomotor movements in late post-operative period (**Video S2**).

Since SP-PBS rats did not walk on the moving treadmill at early period, even when the tail stimulation was added, therefore no percentage change was recorded. At the late period in SP-PBS rats after tail stimulation, the amplitude of movement in the knee joint was also smaller than that in the intact (P<0.05) and in SP-BDNF animals (P<0.05, during early period) (**Figure 4**). However, the amplitude of movement in the ankle joint was variable and not significantly higher than in intact rats. Only the amplitude of movement of the toe joint, which was bigger by approximately 25% (P<0.05) than in intact rats, could partly counterbalance smaller movements in the knee joints. Altogether, these results show that SP-BDNF animals more efficiently counterbalanced the deficit in decreased angular excursion in the knee joint than SP-PBS animals.

### Spinal Cord Transection Leads to the Attenuation of GABA and Glycine Concentration in the Rostral but not in Caudal Lumbar Segments

Little is known on how the content of excitatory and inhibitory neurotransmitters changes long term after complete spinal cord transection in adult rats that do not receive any further treatment and show poor locomotor abilities. We used HPLC to evaluate the segmental levels of neurotransmitter amino acids glutamate, aspartate, GABA and glycine in whole tissue homogenates from adult rats 5 weeks after spinal cord transection (N = 4) and compared them with the levels in intact (N = 4) rats. The levels of all tested amino acids except glutamate were significantly decreased in the lesioned animals [two-way ANOVA: significant differences between Groups (Group F_(1,23)_ = 4,844, P<0.04) and Segments (F_(3,23)_ = 39,191, P<0.000)]. Interactions of Group × Segment were also found (F_(3,23)_ = 6,487, P = 0,002). There was a strong decrease of Asp, Gly and GABA in the lesioned segments (Tukey *post-hoc* test; P = 0.019 (Asp) P = 0.000 (Gly), P = 0.027 (GABA). In the rostral lumbar (L1–2) segments Gly was also significantly decreased (Tukey *post-hoc* test; P = 0.028) whereas GABA tended to decrease (Tukey *post-hoc* test; P = 0.086). No changes were detected in caudal (L3–6) segments of the spinal cord (**Table 3**). These results reveal segmental differences in the responses of neurotransmitters to spinal cord injury and indicate stronger impairment in the inhibitory than excitatory systems in the spinal cord. For the raw data see: **Figure S4** - HPLC.

**Table 3:** The changes of segmental concentration of γ-aminobutyric acid (GABA), glycine (Gly), glutamate (Glu) and aspartate (Asp) measured 5 weeks after complete spinal cord transection in the whole tissue homogenates of thoracic (Th) and lumbar (L) segments.

Neurotransmitter (NT)	Spinal cordsegment	NT concentration(µmol/g wet weight tissue)		Percentage of intact (%)
		Intact rats	Spinal rats	
GABA	Th6–8	0.53±0.076	0.55±0.080	103
	Th9–10 (lesion)	0.53±0.076	0.36±0.093	67*
	L1–2	0.65±0.008	0.49±0.038	76
	L3–6	0.74±0.045	0.79±0.114	108
Gly	Th6–8	1.96±0.176	1.77±0.245	90
	Th9–10 (lesion)	1.96±0.176	1.17±0.258	60***
	L1–2	2.08±0.153	1.53±0.184	74*
	L3–6	2.51±0.167	2.47±0.202	98
Glu	Th6–8	2.01±0.168	1.97±0.258	98
	Th9–10 (lesion)	2.01±0.168	1.60±0.343	80
	L1–2	2.23±0.182	1.87±0.226	84
	L3–6	2.58±0.244	2.68±0.169	104
Asp	Th6–8	0.90±0.106	0.89±0.111	99
	Th9–10 (lesion)	0.90±0.106	0.59±0.155	66*
	L1–2	1.05±0.146	0.83±0.099	80
	L3–6	1.26±0.059	1.42±0.129	113

All samples were measured simultaneously by means of HPLC and the measurements were carried out in quadruplicates. The data of four rats with complete spinal cord transection performed at thoracic segments (Th9–10) and four intact rats are presented. Data show means ± SD, Two way-ANOVA, Tukey *post-hoc* tests, *P<0.05, ***P<0.001.

### BDNF Overexpression in Spinal Rats does not Change Reduced VGluT1 mRNA Expression but Leads to an Increase in VGluT2 mRNA Levels in the Rostral and Caudal Lumbar Segments

There is a strong indication that locomotor training and BDNF increase excitability of motoneurons [8], [19], [35] and activate spinal interneuronal network [36]. However, data are lacking on how the sustained overexpression of BDNF in the lumbar segments affects excitatory and inhibitory interneuronal neurotransmitter systems to achieve treadmill stepping. To determine that, at first we examined gene expression of glutamate vesicular transporters VGluT1 and VGluT2, which reflect predominantly, the activity of glutamatergic neurons in the dorsal spinocerebellar tract (DSCT) in the Clarke’s column (VGluT1 m RNA) [62] and interneurons (VGluT2 mRNA) [63]–[65]. By investigating the levels of mRNA expression, not protein, we could dissociate changes occurring in VGluT1-expressing and VGluT2-expressing glutamatergic interneurons from those related to peripheral and descending glutamatergic tracts.

In intact rats, glutamate vesicular transporter VGluT1 mRNA expression was several times higher in the rostral than in the caudal lumbar segments, in line with the number of DSCT neurons decreasing caudally in the Clarke’s column (**Figure 5A**). After transection, a profound decrease of VGluT1 mRNA was found in the L1–2 (by 95%, Mann-Whitney U test; P = 0.036), but not in L3–6 segments of SP-PBS rats. BDNF overexpression did not affect VGluT1 mRNA levels, which remained low (**Figure 5B**).

In contrast to VGluT1 mRNA, in intact rats there were no segmental differences in the level of VGluT2 expression. After the lesion, a highly significant decrease of VGluT2 mRNA levels was found. Two-way ANOVA revealed a main effect of the animal Group (F_(2,18)_ = 24.21, P<0.000) and interaction of both: Group × Segment (F_(2,18)_ = 9.30, P<0.001). In the L1–2 segments of SP-PBS rats VGluT2 mRNA was decreased by 64% (Tukey *post-hoc* test, P = 0.000) whereas in the L3–6 segments it tended to decrease (by 35%). BDNF overexpression caused VGluT2 mRNA expression increase both in the L1–2 (Tukey *post-hoc* test, P = 0.03) and L3–6 (P = 0.001) segments, where its levels tended to be higher than in intact rats (**Figure 5B**). For the raw data see **Figure S4**– VGluT1/2 mRNA qPCR.

### BDNF Overexpression in Spinal Rats Leads to an Increase in Segmental GABA Concentration with Abnormally High GABA Levels in the Caudal Lumbar Segments

To evaluate the changes in GABA concentration associated with BDNF overexpression, we used ELISA. Spinalization and BDNF overexpression led to significant changes in GABA levels, as revealed by a two-way ANOVA: there was the main effect of the animal Group (F_(2,45)_ = 21,396, P<0.000), and of the Segment (F_(4,45)_ = 15,999, P<0.000), as well as an interaction of Group × Segment (F_(8,45)_ = 4,904, P = 0.000). The 71% decrease in GABA concentration in the transected segment (Tukey *post-hoc* test; P = 0.012) was accompanied by 45% decrease in adjacent segments and 38% decrease in L1–2 segments (n.s.) (**Figure** **6A**).

Overexpression of BDNF did not affect GABA concentration at the lesion site (**Figure 6A**). but led to the normalization of the GABA concentration in the caudal Th and in L1–2 segments. In the L3–6 spinal segments overexpression of BDNF led to highly significant raise in GABA much above levels in SP-PBS rats (Tukey *post-hoc* test; P = 0.000) and that of control (Tukey *post-hoc* test; P<0.001).

### BDNF Overexpression in Spinal Rats Leads to an Increase in Segmental GAD67 mRNA Expression and Immunoreactivity in the Lumbar Segments

An increase in GABA levels caudal to the transection in SP-BDNF group raised the question whether it is due to upregulation of the GABA synthesis. We measured gene expression of two glutamic acid decarboxylase (GAD) enzymes: GAD67, which is responsible for a bulk of neuronal GABA synthesis [66] and GAD65, which is expressed by a more restricted set of interneurons [67].

Two-way ANOVA revealed a main effect of the animal Group F_(2,26)_ = 29,209, P<0.000, and of the Segment (F_(2,26)_ = 23,437, P<0.000), as well as an interaction of both: Group × Segment (F_(4,26)_ = 10,605, P<0.000) on GAD67 mRNA. In the SP-PBS group GAD67 mRNA levels were found to be dramatically reduced in the lesioned thoracic segments (Tukey *post-hoc* test; P = 0,000) and non-significantly reduced in the L1–2 segments, being in line with degree of GABA changes in these segments (**Figure 6B**). Overexpression of BDNF tended to attenuate the GAD67 mRNA decrease in the thoracic segments (Tukey *post-hoc* test; P = 0,081) and led to its rise beyond SP-PBS values in the lumbar segments (P = 0.000), exceeding control values the rostral and caudal lumbar segments (P = 0.05 and P<0.000, respectively; **Figure 6B**), in parallel with GABA overproduction in L3–6 (GAD67 mRNA/GABA correlation at r = 0.853; P<0.05). For the raw data see **Figure S4**– GAD67 mRNA qPCR.

The changes in GAD65 mRNA level demonstrated the same pattern of changes (Two-way ANOVA: a main effect of the animal Group F_(2,16)_ = 32,936, P<0.000, and of the Segment (F_(1,16)_ = 29,532, P<0.000), as well as an interaction of both: Group × Segment (F_(2,16)_ = 9,230, P = 0.002). Tukey *post-hoc* test revealed a significant decrease of GAD65 mRNA level in the rostral lumbar segments after the lesion (P = 0.029). Overexpression of BDNF, on the contrary, led to normalization of GAD65 mRNA expression in the rostral lumbar segments and its strong elevation beyond normal values in the caudal lumbar segments (an increase by 86%, Tukey *post-hoc* test; P = 0.000). For the raw data see **Figure S4**– GAD65 mRNA qPCR.

At the cellular level, we evaluated the BDNF effect on GAD67 protein distribution by immunolabeling performed on sections from SP-BDNF rats. The GAD67 signal was much stronger in the lumbar than in the thoracic segments located rostral to the transection site (**Figure** **6C, D**). In particular, immunolabeling of GAD67 terminals abutting onto motoneuron perikarya was stronger in segments caudal to the transection than rostral to it (**Figure 6C, D**).

To determine the spatial relationship between the regions of higher GAD67 levels and regions with higher BDNF levels, we performed double-immunolabeling for GAD67 and the cMYC-tag. In the regions rich in perikarya and fiber networks expressing the cMYC-tag, GAD67 expression was stronger than in regions poor in cMYC immunolabeling (**Figure 6E, G**). The majority of BDNF/cMYC-expressing perikarya was GAD67-negative (**Figure 6G, H, J**). Altogether, this set of data demonstrates a stimulatory effect of high concentrations of BDNF on the GABAergic system in the spinal cord after transection.

Since the early improvement of locomotor capabilities in SP-BDNF rats was followed by the episodes of hyperactivity despite upregulation of GABAergic, and to a much lesser extent, glutamatergic markers in the caudal lumbar segments, we hypothesized that at this stage the excitatory signaling prevailed. One explanation of this phenomenon could stem from an inefficiency of inhibitory neurotransmission owing to dysfunction of KCC2, Cl^-^ extruder. We therefore asked the question whether in conditions of continuous BDNF overproduction a KCC2 deficit is maintained, thus increasing the possibility of a GABA depolarizing action in our experimental system.

### BDNF Overexpression does not Counteract the Reduced Levels of Potassium-chloride Co-transporter 2 (KCC2) Expression

**Figure 7:**
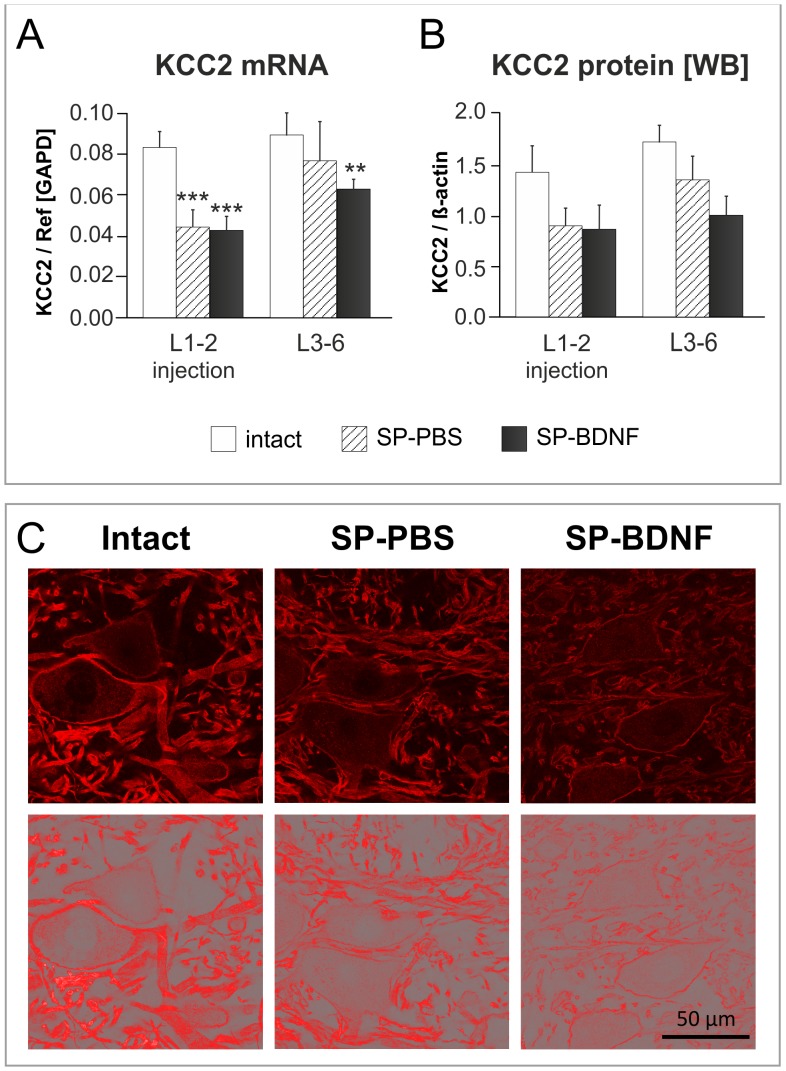
Effect of spinal cord transection and BDNF overexpression on segmental potassium-chloride co-transporter 2 (KCC2) transcript and protein level. (**A**) Spinal cord transection leads to a significant decrease in KCC2 mRNA level in L1–2 segments (hatched bars). In SP-BDNF rats KCC2 mRNA is equally reduced in L1–2 segments and tends to be lower in L3–6 segments than in SP-PBS rats (black bars). (**B**) Similar tendencies were observed at the protein level. Data are the means ± SD (qPCR) or means ± SEM (WB) from 5 intact, 3 SP-PBS and 4 SP-BDNF rats. Two-way ANOVA with Tukey *post-hoc* tests were used; **P<0.01, ***P<0.001. (**C**) The representative confocal microscopy images (upper panel) and the same images tresholded (lower panel) show the pattern and intensity of KCC2 immunostaining of large diameter neurons and surrounding neuropil in the ventral horn of the spinal cord of the rats from intact, SP-PBS and SP-BDNF groups. Note a remarkable reduction of KCC2 labeling in both spinalized groups, with a loss of continuity of the cell membrane signal and torn up appearance of the processes.

In the intact rats KCC2 mRNA and protein were at the same level in the rostral and caudal lumbar segments. Spinal cord transection and BDNF overexpression led to significant changes in KCC2 mRNA. Two-way ANOVA revealed a main effect of the animal Group (F_(2,18)_ = 30,092, P<0.000), and of the Segment (F_(2,18)_ = 21,967, P<0.000) on the transcript level. There was a significant decrease of KCC2 mRNA in L1–2 segments both in SP-PBS rats (by 46%; Tukey *post-hoc* test, P = 0.000) and in the SP-BDNF rats (by 49%; Tukey *post-hoc* test, P = 0.000) as compared to the intact rats (**Figure 7A**). In L3–6 segments the significant decrease was found only in the SP-BDNF group (by 30%; P = 0.007; **Figure 7A**). Interestingly, KCC2 mRNA was correlated negatively with BDNF protein (r = −0.786, P<0.05).

The changes in KCC2 mRNA were accompanied by a similar trend of KCC2 protein levels in both segments (a decrease by 40% and 28%, respectively) (**Figure 7B**; an example of the KCC2 Western blot is shown on **Figure S4**). Two-way ANOVA did show an effect of the Group (F_(2,18)_ = 5,091; P = 0.018) on its level.

The smaller decrease of KCC2 in the caudal lumbar segments than in the rostral ones after the lesion may reflect different degree of denervation and dysfunction of neurons localized proximally and distally to the transection site, which would lead to differentiated impairment in the transcription and, as a result, different protein levels.

Immunofluorescence of KCC2 protein revealed that the corresponding signal, which, in the intact group, was abundant in the membranes of motoneuronal perikarya and in a dense network of thick processes in motor nuclei, was reduced in SP-PBS group, and was decreased further in SP-BDNF animals (**Figure 7C**). Overall, in SP-BDNF group a decrease of KCC2 tended to be more pronounced than in SP-PBS group, suggesting that long-term BDNF overproduction in the spinalized rats augments the deficit in KCC2 caused by the spinal cord transection.

## Discussion

This is the first study correlating the effect of quantified overexpression of BDNF in spinalized rats with segmental changes in excitatory and inhibitory amino acid neurotransmitters, expression of neurotransmitter-related molecules (VGluT1 and VGluT2 transporters and GAD65, GAD67 enzymes) and expression of neuronal excitability-related potassium-chloride co-transporter KCC2. These effects of AAV viral vector mediated BDNF expression translated into robust early improvements in locomotor abilities, suggesting that in principle a stimulation of excitatory circuits in spinal pattern generators could be of clinical relevance. The extent of improvements in body weight support as well as the onset of clonic movements at later time points in conditions of chronic BDNF overexpression, that we detected in our study, are in agreement with the results of the recent study by Boyce and co-workers [36]. Although these authors do not provide any data on the levels of BDNF expression or on the extent of tissue penetration of the neurotrophin, they also found that AAV-BDNF delivery had only a transient beneficial effect. This is a clear indication that enhanced control over BDNF expression by e.g., regulated vector systems is necessary to create the possibility of examination of intraspinal plasticity in conditions of BDNF supply in concentrations more relevant to physiological range.

### Early Improvement of Motor Functions

Evaluation of changes in the treadmill locomotor capabilities of rats after complete transection of the spinal cord and injection of AAV BDNF was done both by means of mBBB scale, introduced by Antri and co-authors [54], [55], and kinematic analysis. The majority of SP-BDNF rats were able to perform alternating steps, place their feet on the planta and support their body weight when their hindlimbs were placed on the moving treadmill. No tactile stimulation of the tail was needed to trigger locomotor movements indicating that excitatory drive delivered by the moving treadmill to the spinal network was sufficient to elicit locomotion of spinal rats overexpressing BDNF. In contrast, SP-PBS rats were not able to walk on the moving treadmill without stimulation of the tail.

Increased excitatory drive to spinal network observed in SP-BDNF rats evolved in time after surgery in some animals as indicated by the differences in response to tactile tail stimulation during treadmill walking in the early and late postoperative periods. SP-BDNF animals attained relatively high scores at the early period (median values 11) but when stimulation of the tail was added during locomotion, their median score rose to 17. In the late testing period they reached the score 16 both without and with tail stimulation. However, approximately 44% of animals improved when the tail stimulation was added but in the other 44% tail stimulation caused deterioration of treadmill locomotion suggesting, that in the latter group the tail stimulation caused hyperexcitation.

Kinematic analysis of treadmill locomotion of SP-BDNF rats revealed that a foot lifting off the treadmill belt was possible due to higher amplitudes in the ankle and toe joints which counterbalanced smaller movements in the knee joints. A similar effect was reported in spinal rats after injection of 8-OHDPAT or quipazine, agonists of 5-HT1A and 5-HT7 receptors [55]. This compensatory effect was diminished in the late post-operative period in SP-BDNF rats, and together with increased frequency of clonic movements attenuated the quality of locomotor movements. Therefore, it is possible that high excitability of the spinal network in SP-BDNF animals, which does not need additional tail stimulation to trigger treadmill locomotion, if controlled with lower BDNF levels, may translate into better quality of locomotor performance.

### The Effectiveness of using the AAV-BDNF

By choosing AAV encoding BDNF under control of the SYN 1 promoter, we secured long-term delivery of proBDNF and mature BDNF forms from neurons. It has been argued that in overexpressing systems the secretion of proBDNF results from overloading the limited capacity of the processing machinery and that under physiological conditions proBDNF is a transient intermediate that is rapidly converted intracellularly to mature BDNF [68]. However, we showed previously, that in the intact rat spinal cord, proBDNF is coexpressed with mBDNF and can be easily detected in the dorsal horn and in motor nuclei [46]. At least in some systems, proBDNF is transported anterogradely, sorted to dense-core synaptic vesicles and released upon depolarization (for discussion see [69]). In our study proBDNF levels were considerably higher in SP-BDNF than in control animals, particularly in the rostral lumbar segments, where also a strong upregulation of expression levels of the pro-proteins convertase - tissue-type plasminogen activator, catalyzing proBDNF extracellular proteolysis, was detected (unpublished data). Taken together, our data may indicate an increase in storage but also in release and extracellular processing of proBDNF. Whereas the role of proBDNF in our experiments remains elusive, it is important that neither apoptotic bodies nor cell nuclei with condensed chromatin in the lumbar segments were observed, which could result from the proapoptotic actions of proBDNF [70], [71].

Some deterioration in gait quality over time could be due TrkB desensitization [72], [73] by BDNF overproduction in the lesioned animals. However, nonphysiological BDNF overexpression did not lead to a further decrease of TrkB protein caused by the lesion and did not downregulate TrkB transcripts [74].

### Spinal Cord Transection Leads to Segmentally Differentiated Changes of BDNF Levels and Excitatory and Inhibitory Amino Acids

Neuronal BDNF levels were reported to either increase or moderately decrease after transection [28], [75] calling into question a pronounced effect of neurotrophin impoverishment on dysfunction of spinal circuits. Quantitation of segmental changes in BDNF levels caused by complete spinal transection in this study showed a tendency of BDNF levels to decrease in the L3–6 segments at 7 weeks post-transection and a profound decrease in BDNF levels in the L1–2 segments, suggesting that the circuits of the rostral lumbar segments are dysfunctional owing to decreased BDNF signaling.

Glutamate is the major excitatory neurotransmitter of interneurons in the lumbar spinal cord circuitry, being essential for intrinsic rhythm generating capability [65]. It also functions in the initiation of locomotion by providing an extrinsic drive to the rhythmogenic circuitry, which in the rat prevails in L1–2 segments [44]. We found a profound deficit in VGluT2 mRNA transporter in the rostral lumbar segments, suggesting that there is severe interneuronal dysfunction and a decrease of excitatory drive from glutamatergic interneurons in the L1–2 circuits after complete spinal cord transection at low-thoracic segments, with smaller impairment of excitatory transmission in the caudal lumbar segments. It is conceivable that perikarya located more rostrally are more vulnerable to transection than those located caudally [76]. In agreement, the reduction of VGluT1 mRNA that is seen predominantly in neurons of the Clarke’s column tract [62], [64] prevails in the rostral lumbar segments with a smaller decrease in caudal segments. To our knowledge, this is the first report on the response of excitatory VGluT2-expressing interneurons following the complete spinal transection at low thoracic segments in adult rats. A moderate decrease of glutamate and aspartate concentrations, which might contribute to this dysfunction, is less diagnostic than reduced VGluT mRNA levels. This is, because, Glu deficits in dysfunctional VGluT1- and VGluT2-expressing neurons may be veiled in the assays of Glu levels done in whole tissue homogenates, by the increased Glu from afferent fibers undergoing reactive synaptogenesis, reported to occur below the level of denervation in adult dogs (for review, see [77]) and rats [17]. When spinal transection was performed in neonatal rats, raised levels of excitation within the lumbar spinal circuitry [19], [78] also suggested reactive sprouting of glutamatergic terminals around motoneuron cell bodies.

A causal link between intrinsic activation of spinal VGluT2-positive neurons located in the lower thoracic and upper lumbar segments and initiation and maintenance of spinal locomotor activity with the appropriate patterns of coordination, has been elegantly evidenced recently in mice [79]. If we assume the same causal link in adult rats, then BDNF-induced recovery of function of VGluT2-positive subpopulation of glutamatergic interneurons, shown in our study, may be sufficient to provide the drive to the spinal network needed to improve locomotion achieved by AAV-BDNF-treated rats. No effect of BDNF on neurons expressing VGluT1 lends support to this notion. Which neuronal subpopulations of VGluT2-positive interneurons are the most affected by the transection and BDNF treatment requires further study.

The decrease of glutamate and aspartate in the rostral lumbar segments was accompanied by a comparable decrease in the concentrations of GABA and glycine, with a tendency of reaching the control levels in the caudal lumbar segments. Since GABA and glycine coexist in the significant fraction of inhibitory terminals in the spinal cord [80]–[82], a comparable contribution of both amino acids to altered inhibitory transmission may be assumed.

The expression of GAD65 and GAD67 revealed the same pattern of changes like GABA. These findings strongly suggest a decrease of inhibition in the rostral, and, to a lesser extent, in the caudal segments in spinal animals and question the proposition that glycinergic and GABAergic inhibition is increased in the caudal lumbar segments [6], [19], [83] contributing to an overall depression of hindlimb movements [21]. The discrepancy between our data and those of others may be related to the time period after transection and species [6], [83]. Indeed, increased glycine levels were reported in neonatally spinalized animals known to recover better their hindlimb function, than adult spinalized animals [84]. However, taking into account that baclofen, agonist of GABA receptor, is a powerful tool in management of spasticity of cerebral and spinal origin, and that spasticity results from hyper-excitability of spinal reflexes in various neurological conditions including spinal cord injury, it is not unreasonable to expect that after injury inhibitory signaling becomes impaired [20]. At the earlier post-lesion time period, one month after spinal cord transection, significant decreases have been shown in the levels and release of GABA and glycine in the ventral grey matter of the lumbar spinal cord of paraplegic dogs (reviewed in [77]). Therefore, involuntary muscle contractions observed in our study may be indicative of enhanced motoneuron excitability in the absence of inhibition from supraspinal systems [85] and inefficiency of intraspinal inhibitory transmission.

### BDNF Overexpression Upregulates Markers of Excitatory and Inhibitory Neurotransmission but does not Eliminate a Deficit of KCC2 Co-transporter

Because the L1–2 segments contained reduced levels of amino acid neurotransmitters after spinalization, enrichment of these segments in BDNF immediately after injury could partially account for an early functional improvement [86], [87]. Also, by providing protection to the surviving neurons and initiating rearrangements of spinal circuitries [88], BDNF could contribute to functional improvement by exerting neurotrophic effects and modulation of synaptic activities [89]–[93]. Our study shows for the first time that a segmental increase in BDNF concentration leads to an increase in GABA content, and in GAD65 and GAD67 expression in the lumbar segments. GAD67 labeling spatially correlated with BDNF-transgene expression. With this respect it is worth to mention that the neuronal PAS domain protein 4 (Npas4), a transcriptional factor which specifically controls the activity-dependent BDNF mRNA levels, controls also GABAergic synapse development, affecting GAD65/67 and GABAergic receptor expression [94].

Additionally to increased expression of enzymes which control GABA content, an increase of GAD67 in the terminals around motoneuronal perikarya indicated that the inhibitory inputs to motoneurons were strengthened. This is in agreement with results showing that BDNF treatment mainly support sprouting of F-type boutons with presumably inhibitory function [95]. Considering the functional consequences of these changes, it is note-worthy that abnormally high glutamate and glycine levels in the lumbar spinal cord in trained spinalized rats correlated with the ability to perform independent stepping [19]. We can ask the question whether in conditions of a permanent loss of the supraspinal control, locomotor stepping requires an elevation of both excitatory and inhibitory signaling from the remaining circuits above controls. Abnormally high levels of GABA accompanied by an elevated expression of VGluT2 mRNA were found in the caudal lumbar segments in SP-BDNF-treated rats in the present study, supporting this reasoning. Importantly, increased GABA levels may modulate responses to injury by preventing macrophage/microglia activation, inhibiting pro-inflammatory cytokine release by glial cells [96]–[100] and by reducing oxygen consumption and blood flow [101].

At the late time points (6–7 weeks) upregulation of GABAergic markers of interneuronal circuits was more pronounced than upregulation of VGluT2 glutamatergic marker. Despite that, episodes of increased myoclonus, which is attenuable by drugs augmenting GABAergic transmission [102], and which developed over time in lesioned animals treated with AAV-BDNF, suggested that excitatory signaling prevailed. How do we interpret this shift towards increased intrinsic excitability in a context of changes in GABAergic system?

A decrease of KCC2 co-transporter, was first documented in the ventral horn of spinal rats [43], and confirmed in this study. Such decrease might reduce the generation of Cl^-^ dependent hyperpolarizing postsynaptic currents mediated by ionotropic GABA(A) and glycine receptors [103]–[105], particularly in conditions of increased inhibitory signaling. Such conditions were found in the caudal lumbar segments of AAV-BDNF treated rats. Thus, concomitantly with a persisting reduction of KCC2 levels, shown to increase spontaneous neuronal activity and to accelerate locomotor-like activity [106], the probability of reversal of inhibitory signaling by GABA, and possibly also glycine, is increased. It may contribute to depolarization instead of hyperpolarization [40], [107], leading to increased intrinsic excitability. It is note-worthy that single intrathecal BDNF injection two weeks after spinal cord transection was reported to increase KCC2 in the plasma membranes in the lumbar spinal cord a day after injection [43], that may be indicative, again, of the necessity to introduce regulated vector system to optimize effects of BDNF treatment.

The changed excitability of the spinal network after spinalization is a phenomenon shown to engage also other neurotransmission-related molecules. Wienecke and coworkers [108] showed that after transection of the caudal spinal cord the NMDA receptor complex is up-regulated while GABA(A) receptor is down-regulated. Complete midthoracic transection at P5 results in a long-term deficit of GABA(A) γ2 subunit levels in the soleus motoneurons [109], suggesting impairment of GABA signaling in this motoneuron pool, which is also impoverished in cholinergic innervation after spinal cord transection in adult rats [110]. Of particular importance are serotonin 2A and 2C receptors (5-HT-2A and 5HT-2C) which are likely the most important 5HT receptors for regulating the motoneuron excitability. Therefore it is worth to mention that in the late period (45 days) after sacral spinal transection upregulation of 5HT-2C receptors in the motoneuron somata develops [111], positively correlating with development of spasticity. Paradoxically, activation of 5-HT-2A receptors has been recently shown [112] to increase the cell membrane expression of KCC2, to restore endogenous inhibition and reduce spasticity after spinal cord transection at low-thoracic segments. These data indicate the complexity of the mechanisms which lead to altered motoneuron excitability.

**Figure 8:**
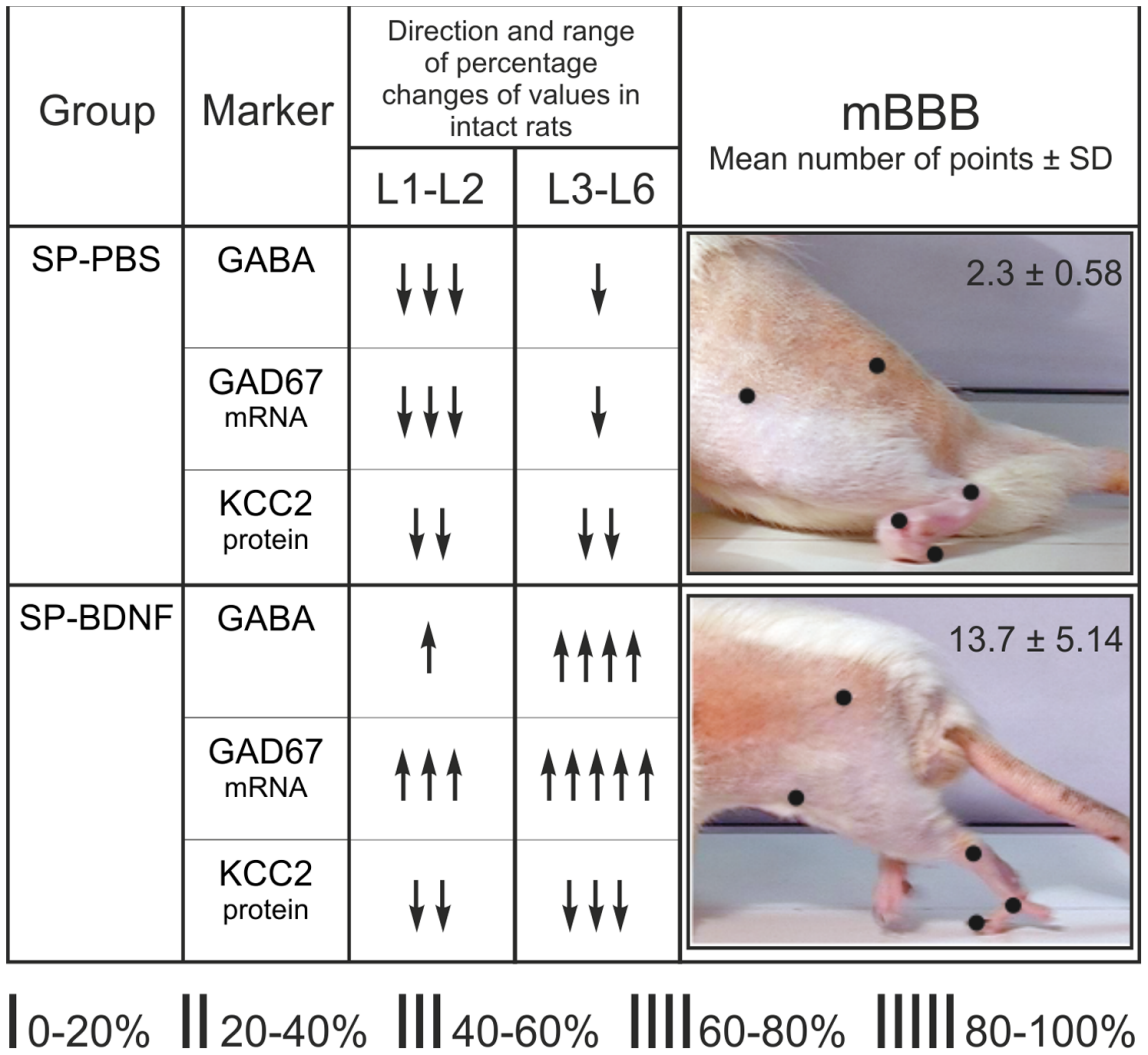
Summary of the effect of complete spinal cord transection and BDNF overexpression in spinal rats on the levels of GABA and transcripts of GAD67 and KCC2 in the rostral and caudal segments of the lumbar spinal cord in the late post-operative period, in conjunction with the scores of their locomotor performance with no tail stimulation, on the moving treadmill.

Since the changes in motoneurons that resulted from a removal of voluntary drive have been reported [113], we propose that rats overexpressing BDNF require less synaptic drive to motoneurons to elicit weight-bearing locomotor movements than control rats. This interpretation is in line with data showing the reduced threshold in activity of motoneurons in AAV-BDNF-treated rats versus control rats [36]. We propose that maintenance of a KCC2 deficit in BDNF overexpressing rats, in conjunction with normal GABA levels in L1–2 segments and elevated GABA levels in L3–6 segments (summarized in **Figure 8**) strengthen inhibitory inputs of the lumbar circuitries and thus render hindlimb motoneurons more excitable.

In summary, long-term enrichment of the isolated spinal networks in BDNF significantly improved plantar stepping which started shortly after complete transection. The symptoms of hyperexcitability developing over time might be due to a progressing disturbance of the balance between excitation and inhibition of motoneurons by interneurons with lesion-induced alterations of their properties.

## Supporting Information

Figure S1 **Overexpression of recombinant BDNF in primary cells in cortico-hippocampal cultures transduced with AAV1/2 viral vector results in the production of biologically active protein.** (**A**) A representative Western blot demonstrating pro-BDNF and mBDNF expression in control, untreated primary cells, control cells treated with conditioned media (30 min) and BDNF-transduced cells 6 days after transduction (DIV 12). (**B**) The kinetics of BDNF secretion into the culture media from cells transduced with the BDNF transgene (ELISA). The culture media from non-transduced cells served as the control. (**C**) Western blot analysis of TrkB signaling in control, untreated primary cells, and primary cells treated with conditioned media (30 min). In all Western blot experiments (**A, C**) the treatment of control cells with human recombinant BDNF protein (50 ng/mL) served as a positive control.
(TIF)

Figure S2 **Macroscopic analysis of the lesion completeness.** To verify the completeness of the spinal cord transection the macroscopic analysis was performed. The photographs were taken immediately after spinal cords were dissected from the vertebral columns. Good reproducibility of the lesion and injection site is documented.
(TIF)

Figure S3 **Immunohistochemical staining for serotonergic (5HT) fibers for the verification of the lesion completeness.** To verify the completeness of the spinal cord transection immunohistochemical staining for serotonergic (5HT) fibers was performed seven weeks after operation. Since spinal 5HT fibers constitute a projection descending from the raphe nuclei, a lack of 5HT-immunoreactivity in the spinal cord segments below the lesion site is a strong indication of complete isolation of these segments from the supraspinal structures. The example shown is representative for all other animals with transection which were analyzed in this study.
(TIF)

Figure S4 **Raw data from the HPLC and real-time quantitative RT-PCR analysis and an example of the KCC2 Western blot experiment.**
(PDF)

Table S1 **Real-time PCR amplicons for relative quantification of BDNF, GAD67, GAD65, KCC2, VGluT1 and VGluT2 expression levels.**
(DOCX)

Video S1 **Treadmill locomotion of adult rats after complete spinal cord transection at early post-surgery period; comparison between PBS- and AAV-BDNF injected subjects.**
(MPG)

Video S2 **Treadmill locomotion of adult rats after complete spinal cord transection at late post-surgery period; comparison between PBS- and AAV-BDNF- injected subjects.** The film presents also the episodes of the musculature spasms occurring in AAV-BDNF injected rat.
(MPG)
