# Correction: Overexpression of BDNF Increases Excitability of the Lumbar Spinal Network and Leads to Robust Early Locomotor Recovery in Completely Spinalized Rats

**DOI:** 10.1371/journal.pone.0092439

**Published:** 2014-03-24

**Authors:** The PLOS ONE Staff

A funder is incorrectly omitted from the Funding statement. The correct Funding statement is as follows:

This work was supported by a Polish-German cooperation grant (S007/P-N/2007/01); National Science Center grant N N401 324739; EMBO Short-Term Fellowship to E. Z. (ASTF 211.00.2007); GA No 264173 (Bio-Imagine) and statutory funds for the Nencki Institute. The funders had no role in study design, data collection and analysis, decision to publish, or preparation of the manuscript.
