# Supplementary figures and images for: Overexpression of BDNF Increases Excitability of the Lumbar Spinal Network and Leads to Robust Early Locomotor Recovery in Completely Spinalized Rats
Source: PLoS One. 2014 Feb 14;9(2):e88833. doi: 10.1371/journal.pone.0088833 (PMC3925164; doi:10.1371/journal.pone.0088833)

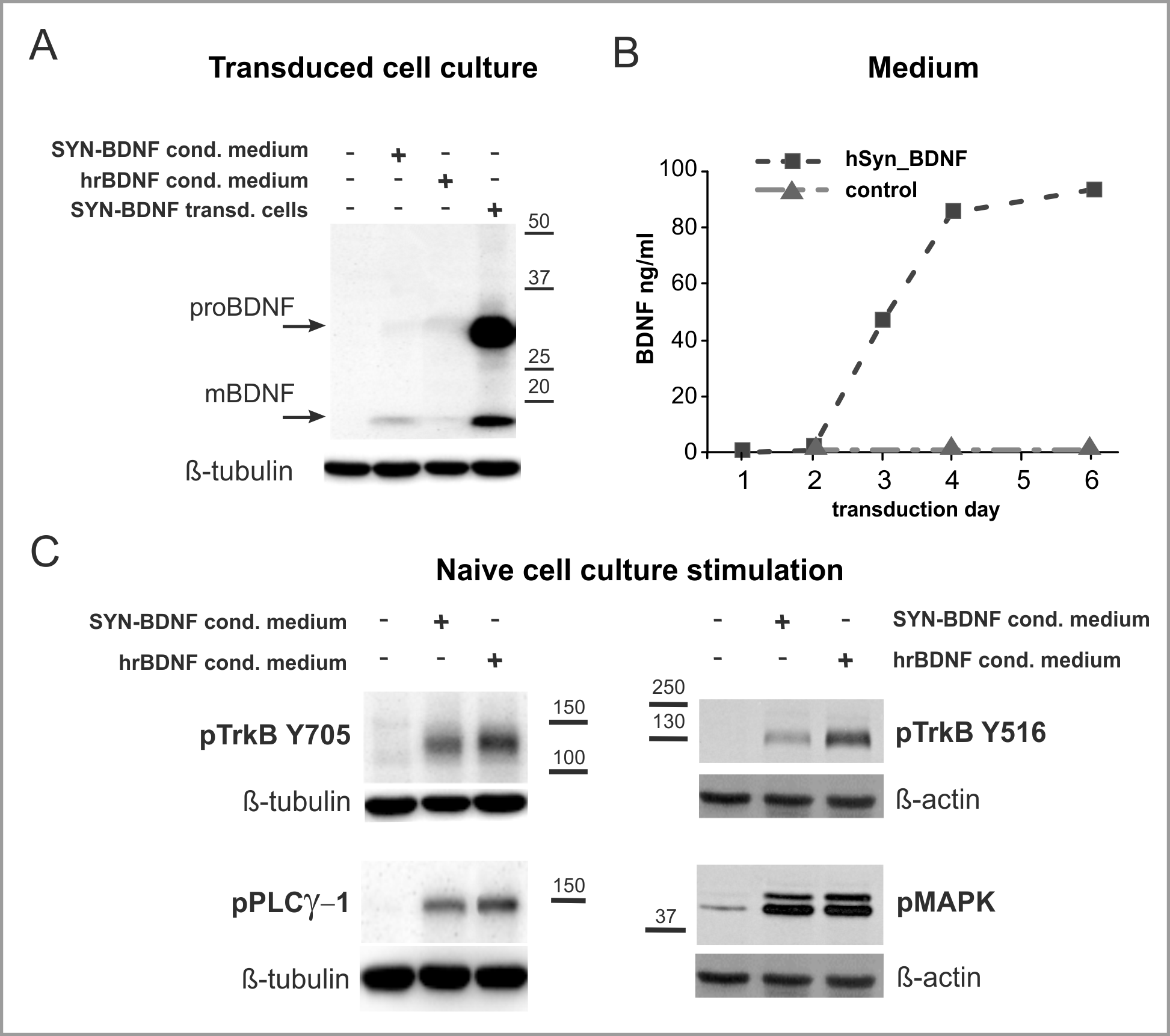

Supplement: Figure S1 — Overexpression of recombinant BDNF in primary cells in cortico-hippocampal cultures transduced with AAV1/2 viral vector results in the production of biologically active protein. (A) A representative Western blot demonstrating pro-BDNF and mBDNF expression in control, untreated primary cells, control cells treated with conditioned media (30 min) and BDNF-transduced cells 6 days after transduction (DIV 12). (B) The kinetics of BDNF secretion into the culture media from cells transduced with the BDNF transgene (ELISA). The culture media from non-transduced cells served as the control. (C) Western blot analysis of TrkB signaling in control, untreated primary cells, and primary cells treated with conditioned media (30 min). In all Western blot experiments (A, C) the treatment of control cells with human recombinant BDNF protein (50 ng/mL) served as a positive control. (TIF) [file pone.0088833.s001.tif]

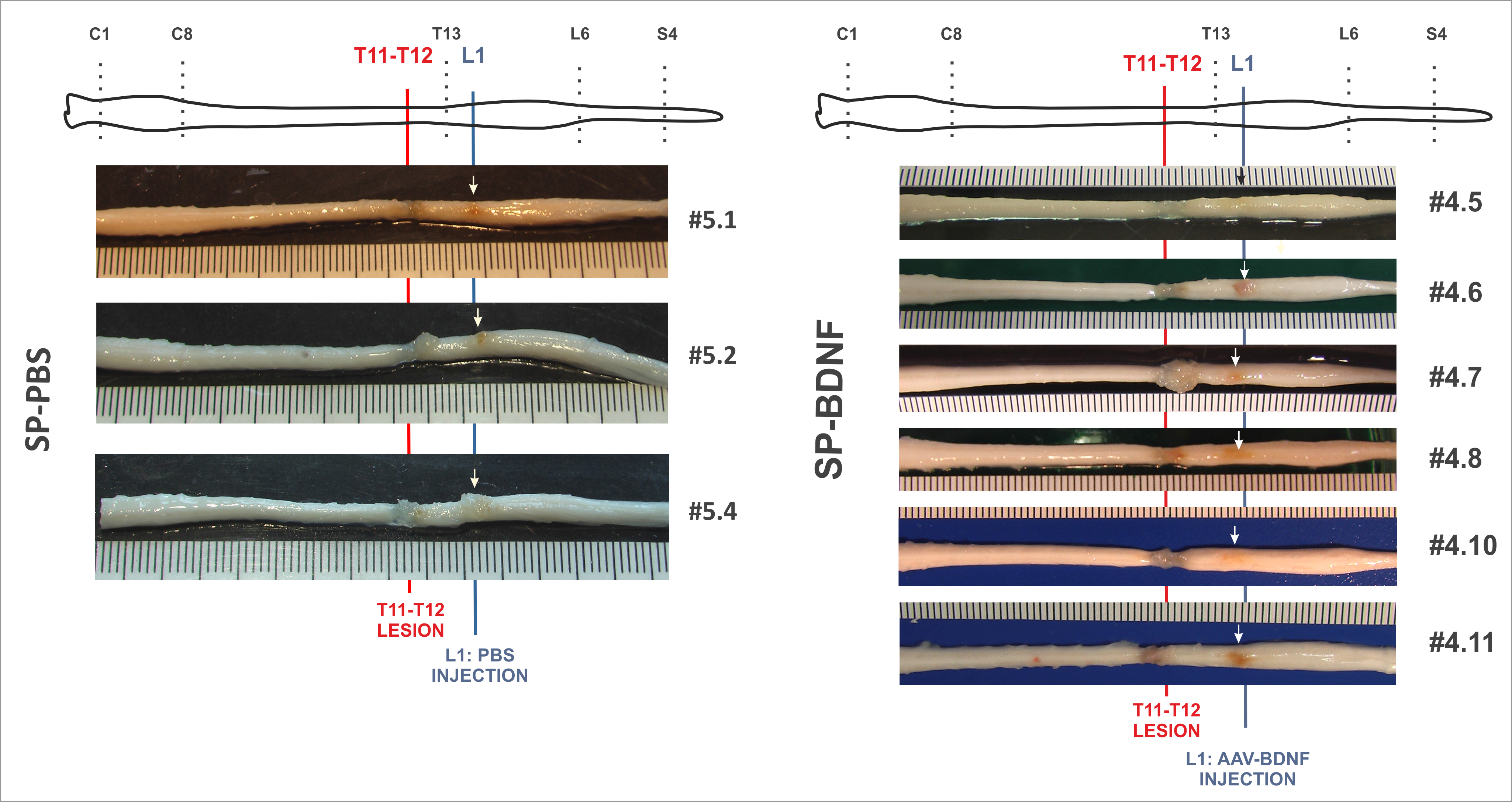

Supplement: Figure S2 — Macroscopic analysis of the lesion completeness. To verify the completeness of the spinal cord transection the macroscopic analysis was performed. The photographs were taken immediately after spinal cords were dissected from the vertebral columns. Good reproducibility of the lesion and injection site is documented. (TIF) [file pone.0088833.s002.tif]

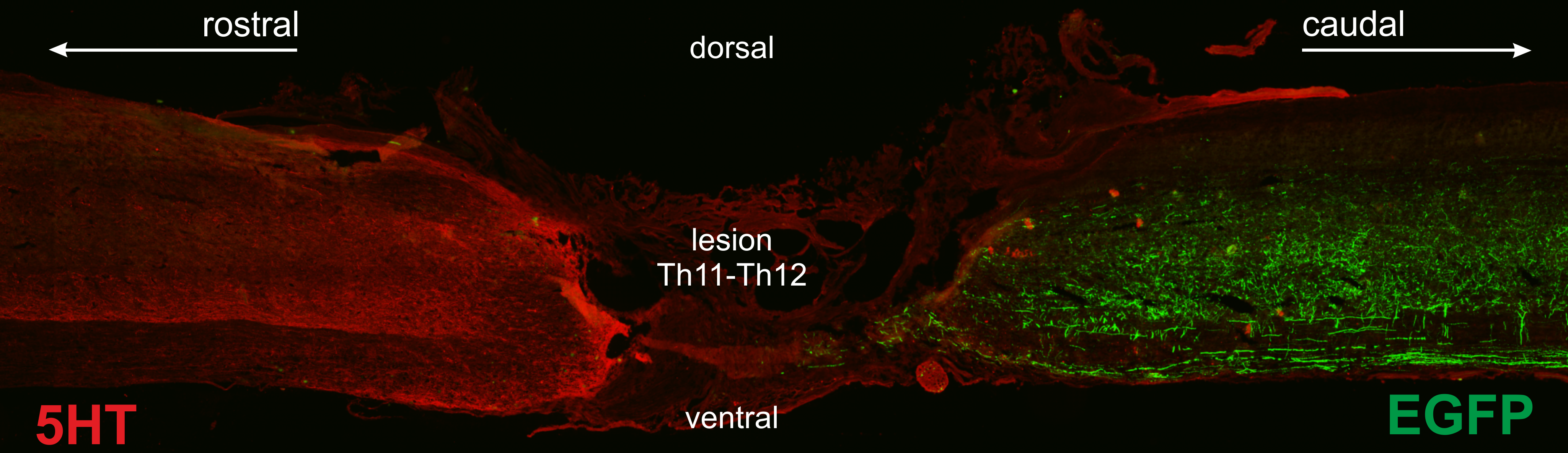

Supplement: Figure S3 — Immunohistochemical staining for serotonergic (5HT) fibers for the verification of the lesion completeness. To verify the completeness of the spinal cord transection immunohistochemical staining for serotonergic (5HT) fibers was performed seven weeks after operation. Since spinal 5HT fibers constitute a projection descending from the raphe nuclei, a lack of 5HT-immunoreactivity in the spinal cord segments below the lesion site is a strong indication of complete isolation of these segments from the supraspinal structures. The example shown is representative for all other animals with transection which were analyzed in this study. (TIF) [file pone.0088833.s003.tif]
