# Supplementary material for: Overexpression of BDNF Increases Excitability of the Lumbar Spinal Network and Leads to Robust Early Locomotor Recovery in Completely Spinalized Rats
Source: PLoS One. 2014 Feb 14;9(2):e88833. doi: 10.1371/journal.pone.0088833 (PMC3925164; doi:10.1371/journal.pone.0088833)
Supplement: Figure S4 — Raw data from the HPLC and real-time quantitative RT-PCR analysis and an example of the KCC2 Western blot experiment. (PDF) [file pone.0088833.s004.pdf]

HPLC data

AA - GABA [umol/g wet tissue]

Rat number	Th 6-8	Th 9-10 (lesion)	L1-2	L3-6
Control 1	0,55996	0,55996	0,64896	0,80005
Control 2	0,46118	0,46118	0,64343	0,73052
Control 3	0,62951	0,62951		0,69266
Control 4	0,48802	0,48802	0,65913	0,72396
Spinal 1	0,61786	0,25709	0,47737	0,88966
Spinal 2	0,54459	0,46636	0,54122	0,89825
Spinal 3	0,59783	0,40293	0,45147	0,69637
Spinal 4	0,43924	0,31548	0,50520	0,69534

AA - Glycin [umol/g wet tissue]

Rat number	Th 6-8	Th 9-10 (lesion)	L1-2	L3-6
Control 1	1,98962	1,98962	2,17749	2,59370
Control 2	1,83503	1,83503	1,90143	2,68863
Control 3	2,19716	2,19716		2,30644
Control 4	1,81867	1,81867	2,15510	2,44951
Spinal 1	1,92006	0,97632	1,61825	2,74686
Spinal 2	1,73465	1,12777	1,27126	2,45054
Spinal 3	1,98142	1,53347	1,54125	2,42790
Spinal 4	1,43610	1,03252	1,69440	2,26176

AA - Glutamic acid [umol/g wet tissue]

Rat number	Th 6-8	Th 9-10 (lesion)	L1-2	L3-6
Control 1	2,03058	2,03058	2,12076	2,49303
Control 2	1,79055	1,79055	2,13196	2,88896
Control 3	2,20015	2,20015		2,30855
Control 4	2,03163	2,03163	2,44122	2,62825
Spinal 1	2,19212	1,22380	1,61490	2,88664
Spinal 2	1,81540	1,61919	1,79557	2,74925
Spinal 3	2,19454	2,05229	2,14898	2,56468
Spinal 4	1,69412	1,52390	1,93840	2,52134

AA - Aspatrate [umol/g wet tissue]

Rat number	Th 6-8	Th 9-10 (lesion)	L1-2	L3-6
Control 1	0,97387	0,97387	1,03179	1,31970
Control 2	0,78695	0,78695	0,90984	1,22159
Control 3	0,99919	0,99919		1,20362
Control 4	0,82446	0,82446	1,20081	1,30727
Spinal 1	0,91769	0,41911	0,80295	1,56775
Spinal 2	0,79724	0,55194	0,72830	1,39896
Spinal 3	1,03586	0,79436	0,96630	1,46694
Spinal 4	0,80861	0,58443	0,83529	1,26147

Intact control
Spinal

GABA [umol/100mg protein]

**Rat number**	Th rost lesion	Th 11-12 (lesion)	Th caud lesion	L1-2 (injection)	L3-6
7.1	2,04215	2,04215	2,04215	1,92074	3,01310
7.2	2,17031	2,17031	2,17031	2,46081	2,06290
7.3	1,96020	1,96020	1,96020	2,17879	1,74621
7.4	1,85779	1,85779	1,85779	2,10936	2,59679
7.5	3,08792	3,08792	3,08792	3,20642	3,30886
5.1	0,83246	0,18554	0,75297	0,85715	1,36801
5.2	1,16628	0,93624	1,10339	1,36792	2,54127
5.4	1,82951	0,78454	1,61855	1,99339	2,50181
4.6	0,96575	0,71581	2,21004	2,20886	4,39760
4.8	1,51804	1,66535	1,88369	2,01586	4,85842
4.10	1,59547	0,65423	2,07206	3,13199	4,27524
4.11	2,08804	1,62570	3,09959	3,19556	3,71653

Intact control
SP-PBS
SP-BDNF

BDNF [ng/100mg protein]

**Rat number**	Th rost lesion	Th 11-12 (lesion)	Th caud lesion	L1-2 (injection)	L3-6
7.1	194,68	194,68	194,68	149,52	108,40
7.2	292,64	292,64	292,64	192,29	200,51
7.3	217,93	217,93	217,93	129,25	120,87
7.4	229,84	229,84	229,84	142,54	182,16
7.5	235,04	235,04	235,04	181,27	160,40
5.1	70,27	47,47	85,20	121,20	113,20
5.2	104,31	195,19	78,56	89,63	168,45
5.4	104,23	96,18	88,00	93,41	139,51
4.6	66,88	9026,40	14898,17	18441,15	13645,41
4.8	94,91	5818,67	48554,58	43970,32	29668,18
4.10	146,56	11538,08	18116,95	40340,52	17919,49
4.11	56,42	13904,69	16617,19	34773,92	12230,24

Intact control
SP-PBS
SP-BDNF

BDNF/GAPDH mRNA

**Rat number**	Th 11-12 (lesion)	L1-2 (injection)	L3-6
7.1	0,0082	0,0096	0,0085
7.2	0,0060	0,0080	0,0065
7.3	0,0055	0,0098	0,0087
7.4	0,0066	0,0064	0,0064
7.5	0,0069	0,0060	0,0092
5.1	0,0006	0,0033	0,0049
5.2	0,0015	0,0021	0,0069
5.4	0,0024	0,0018	0,0030
4.6	0,0377	8,9785	0,3912
4.8	0,0172	7,8970	0,4999
4.10	0,0159	3,8370	0,4587
4.11	0,0204	3,5555	0,1541

Intact control
SP-PBS
SP-BDNF

GAD67/GAPDH mRNA

**Rat number**	Th 11-12 (lesion)	L1-2 (injection)	L3-6
7.1	0,1381	0,1157	0,0935
7.2	0,1103	0,1488	0,1106
7.3	0,1022		0,1054
7.4	0,1198	0,1053	0,1098
7.5	0,1283	0,0814	0,1254
5.1	0,0017	0,0785	0,1330
5.2	0,0092	0,0558	0,0821
5.4	0,0034	0,0479	0,0824
4.6	0,1073	0,2236	0,2513
4.8	0,0563	0,1541	0,2563
4.10	0,0053	0,1739	0,2059
4.11	0,0669	0,1741	0,1472

Intact control
SP-PBS
SP-BDNF

GAD65/GAPDH mRNA

**Rat number**	L1-2 (injection)	L3-6
7.1	0,0644	0,0616
7.2	0,0700	0,0803
7.3	0,0816	0,0764
7.4	0,0732	0,0806
7.5	0,0510	0,0649
5.1	0,0500	0,0726
5.2	0,0304	0,0530
5.4	0,0284	0,0560
4.8	0,0527	0,1428
4.10	0,0898	0,1426
4.11	0,0853	0,1216

Intact control
SP-PBS
SP-BDNF

KCC2/GAPDH mRNA

**Rat number**	L1-2 (injection)	L3-6
7.1	0,0943	0,0767
7.2	0,0833	0,0892
7.3	0,0843	0,0967
7.4	0,0817	0,1016
7.5	0,0743	0,0836
5.1	0,0535	0,0982
5.2	0,0447	0,0627
5.4	0,0376	0,0669
4.6	0,0496	0,0671
4.8	0,0381	0,0587
4.10	0,0370	0,0669
4.11	0,0471	0,0586

Intact control
SP-PBS
SP-BDNF

VGluT1/GAPDH mRNA

**Rat number**	L1-2 (injection)	L3-6
7.1	0,01491	0,00088
7.2	0,01271	0,00073
7.3	0,01599	0,00081
7.4	0,01290	0,00304
7.5	0,01023	0,00108
5.1	0,00066	0,00024
5.2	0,00036	0,00047
5.4	0,00082	0,00100
4.6	0,00087	0,00046
4.8	0,00030	0,00088
4.10	0,00113	0,00060
4.11	0,00213	0,00040

Intact control
SP-PBS
SP-BDNF

VGluT2/GAPDH mRNA

**Rat number**	Th 11-12 (lesion)	L1-2 (injection)	L3-6
7.1	0,0793	0,1310	0,0704
7.2	0,0842	0,1162	0,0817
7.3	0,0826	0,1247	0,0865
7.4	0,0751	0,1172	0,1016
7.5	0,0949	0,0976	0,1100
5.1	0,0006	0,0563	0,0697
5.2	0,0048	0,0364	0,0516
5.4	0,0007	0,0343	0,0547
4.6	0,0210	0,0887	0,1427
4.8	0,0281	0,0560	0,1359
4.10	0,0320	0,0888	0,1081
4.11	0,0172	0,1052	0,0999

Intact control
SP-PBS
SP-BDNF

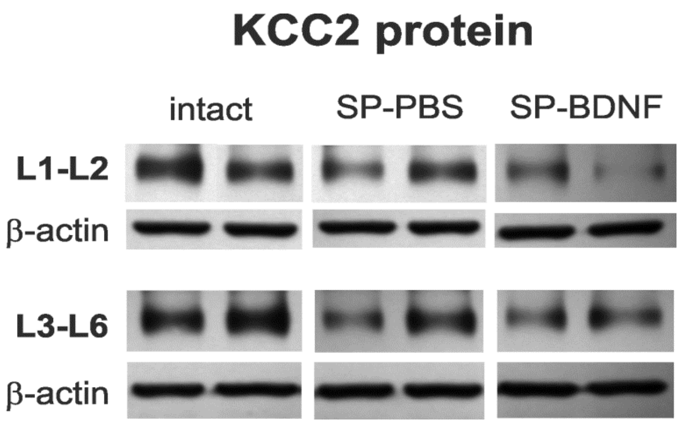

Ziemlińska et al. (2013) Overexpression of BDNF increases excitability of the lumbar spinal network and leads to robust early locomotor recovery in completely spinalized rats
