# Supplementary material for: Overexpression of BDNF Increases Excitability of the Lumbar Spinal Network and Leads to Robust Early Locomotor Recovery in Completely Spinalized Rats
Source: PLoS One. 2014 Feb 14;9(2):e88833. doi: 10.1371/journal.pone.0088833 (PMC3925164; doi:10.1371/journal.pone.0088833)
Supplement: Table S1 — Real-time PCR amplicons for relative quantification of BDNF, GAD67, GAD65, KCC2, VGluT1 and VGluT2 expression levels. (DOCX) [file pone.0088833.s005.docx]

| **Marker** [UPL probe number] | **Real-time PCR amplicon [**5’ PRIMER – ***UPL PROBE*** – PRIMER 3’**]** |
| --- | --- |
| Accession number in NCBI or Ensemble database |  |
| **BDNF** [#67] | GCAGTCAAGTGCCTTTGGAGCCTCCTCTGCTCTTTC***TGCTGGAG***GAATACAAAAATTACCTGGATGCCG |
| NM_012513.3 |  |
| **GAD67** [#129] | CCGCCATGTGGACATCTTCAAG***TTCTGGCT***GATGTGGAAAGCAAAGGGCACTGTGGGATTTGAAAATCAGATCAACAAATGCCTGG |
| NM_017007.1 |  |
| **GAD65** [#129] | GAGCCGCCTCTCAAAGGTGGCGCCAGTGATTAA***AGCCAGAA***TGATGGAGTATGGGACCACAATGG |
| NM_012563.1 |  |
| **KCC2** [#75] | TTCTGGACAACCACCCTCAGGCTCAGCGGGCAGAGGAGTCTATCCGGCGCCTGAT***GGAGGCTG***AGAAGGTGAAGGGCTTCTGCC |
| U55816.1 |  |
| **VGluT1** [#75] | GCAGGAGGAAGTTTCGGAAGCTGGCGGGGCGCGCCCTGG***GGAGGCTG***CACCGGTTACTGGAGAAGCGGCAGGAAGGCGCGGAGACATTGGAGCTGAGCG |
| ENSRNOT00000028064.2 |  |
| **VGluT2** [#67] | GGAAAATCCCTCGGACAGATCTACAGGG***TGCTGGAG***AAGAAGCAGGATAACCGAGAGACCATCGAGC |
| ENSRNOT00000022383.4 |  |
